# Boosted Photocatalytic Performance for Antibiotics Removal with Ag/PW_12_/TiO_2_ Composite: Degradation Pathways and Toxicity Assessment

**DOI:** 10.3390/molecules28196831

**Published:** 2023-09-27

**Authors:** Hongfei Shi, Haoshen Wang, Enji Zhang, Xiaoshu Qu, Jianping Li, Sisi Zhao, Huajing Gao, Zhe Chen

**Affiliations:** 1 Institute of Petrochemical Technology, Jilin Institute of Chemical Technology, Jilin City 132022, China; 18638813901@163.com (H.W.); 13664448948@163.com (E.Z.); xiaoshuqu@jlict.edu.cn (X.Q.); huajing_gao@163.com (H.G.); chenzhecz999@163.com (Z.C.); 2 Institute of Catalysis for Energy and Environment, College of Chemistry & Chemical Engineering, Shenyang Normal University, Shenyang 110034, China; zhaoss0905@163.com

**Keywords:** Ag nanoparticles, PW_12_/TiO_2_ nanofibers, degradation of antibiotics, degradation pathways, toxicity assessment

## Abstract

Photocatalyst is the core of photocatalysis and directly determines photocatalytic performance. However, low quantum efficiency and low utilization of solar energy are important technical problems in the application of photocatalysis. In this work, a series of polyoxometalates (POMs) [H_3_PW_12_O_40_] (PW_12_)-doped titanium dioxide (TiO_2_) nanofibers modified with various amount of silver (Ag) nanoparticles (NPs) were prepared by utilizing electrospinning/photoreduction strategy, and were labelled as *x* wt% Ag/PW_12_/TiO_2_ (*abbr. x*% Ag/PT, *x* = 5, 10, and 15, respectively). The as-prepared materials were characterized with a series of techniques and exhibited remarkable catalytic activities for visible-light degradation tetracycline (TC), enrofloxacin (ENR), and methyl orange (MO). Particularly, the 10% Ag/PT catalyst with a specific surface area of 155.09 m^2^/g and an average aperture of 4.61 nm possessed the optimal photodegradation performance, with efficiencies reaching 78.19% for TC, 93.65% for ENR, and 99.29% for MO, which were significantly higher than those of PW_12_-free Ag/TiO_2_ and PT nanofibers. Additionally, various parameters (the pH of the solution, catalyst usage, and TC concentration) influencing the degradation process were investigated in detail. The optimal conditions are as follows: catalyst usage: 20 mg; TC: 20 mL of 20 ppm; pH = 7. Furthermore, the photodegradation intermediates and pathways were demonstrated by HPLC-MS measurement. We also investigated the toxicity of products generated during TC removal by employing quantitative structure-activity relationship (QSAR) prediction through a toxicity estimation software tool (T.E.S.T. Version 5.1.2.). The mechanism study showed that the doping of PW_12_ and the modification of Ag NPs on TiO_2_ broadened the visible-light absorption, accelerating the effective separation of photogenerated carriers, therefore resulting in an enhanced photocatalytic performance. The research provided some new thoughts for exploiting efficient and durable photocatalysts for environmental remediation.

## 1. Introduction

In recent years, photocatalysis technology, which can use solar energy for environmental purification and energy conversion, has received worldwide attention [1,2]. Photocatalytic technology has a wide range of applications in pollutants degradation, CO_2_ reduction, water splitting to produce hydrogen and nitrogen fixation, etc. [3]. The core of photocatalysis is designing and developing the photocatalysts with visible-light response, prominent catalytic activity, and recyclability. Among the various photocatalysts, TiO_2_ has received a lot of attention due to its low synthesis cost, lack of toxicity, and high catalytic activity [4]. However, the wide band gap and low utilization efficiency of carriers limit its practical applications [5]. Therefore, it is urgent to enhance the visible-light absorption and the driving force for the separation of photoinduced carriers. Many strategies have been made to improve its catalytic activity, including dye sensitization [6], construction of heterojunction [7], morphology engineering [8], and metal/non-metal element doping, etc. [9].

POMs are identified as a promising candidate to embellish TiO_2_ for addressing this challenge. POMs demonstrate semiconductor-like characteristics with their tunable electronic structures and energy levels. They also possess high negative charge and excellent solubility and are endowed with favorable processing properties [10,11]. Therefore, POMs are easily encapsulated or dispersed within various semiconductors, which can constantly enhance the redox property, modulate the band gap structure, and facilitate the separation efficiency of photoproduced carriers [12,13,14]. Among various POMs, H_3_PW_12_O_40_ (*abbr.* PW_12_), as a Keggin-type POM, has demonstrated important applications in photocatalysis fields such as water splitting and contaminants removal [15,16].

Besides, the strategy of noble metals (such as Ag, Pd, Pt, and Au) modifying semiconductors has been extensively investigated to expand spectral absorption and accelerate the separation of photon-generated carriers [17,18,19]. Typically, a Schottky junction is formed at the interface between a metal and a semiconductor to create a built-in electric region that enhances the surface plasmon resonance (SPR) effect. Among these noble metals, Ag has been extensively applied in SPR photocatalysis due to the excellent electrical conductivity, relatively cheap price, wide SPR absorption, and intense local electromagnetic fields caused by SPR [20,21]. For instance, Ag@TiO_2_ composites with core-shell nanostructures were prepared, applying the one-step solvothermal method by Zeng et al., which displayed enhanced light absorption range and enabled the effective separation of e^−^-h^+^ pairs, resulting in an improved photocatalytic performance [22]. Moreover, the electrostatic spinning technology has been considered as a versatile technology capable of adjusting the composition, diameter, and orientation of materials according to the intended function and application [23], which is employed extensively in the fabrication of metal oxides (TiO_2_, ZnO, Fe_2_O_3_, WO_3_, etc.) nanofibers for photocatalytic degradation of pollutants [24], hydrogen production [25], and CO_2_ reduction [26], etc.

Based on the above considerations, we prepared a novel Ag/PW_12_/TiO_2_ (*abbr.* Ag/PT) composite by electrospinning/photoreduction methods, according to the literature [11,19]. Firstly, the electrospinning/calcination method was used to obtain PW_12_/TiO_2_ material; then, the Ag NPs were loaded on PW_12_/TiO_2_ using the photoreduction method, obtaining the Ag/PT composite. Moreover, these as-prepared Ag/PT nanofibers exhibited remarkable photocatalytic activities for the degradation of multiple pollutants. The 10% Ag/PT catalyst possessed the optimal photodegradation performance, whose efficiency reached 78.19% for TC, 93.65% for ENR, and 99.29% for MO, which was significantly higher than those of PW_12_-free Ag/TiO_2_ and PT. Furthermore, the influence parameters, including the pH of the solution, catalyst usage, and the concentration of TC, were studied in detail. The degradation intermediates and pathways were revealed by LC-MS data. QSAR prediction was employed to investigate the toxicity of products in TC photodegradation. Ultimately, the photocatalytic mechanism was investigated with radical capture analysis and band gap structures.

## 2. Results and Discussion

### 2.1. Characterization of Ag/PT Composites

**Figure 1:**
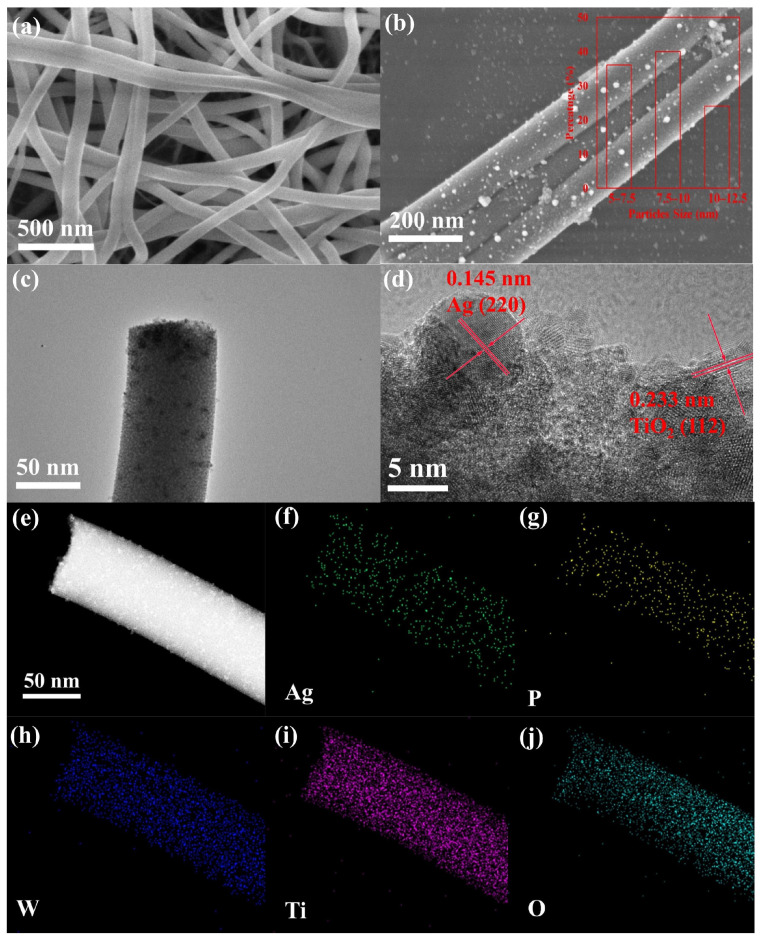
SEM images of PT (**a**) and 10% Ag/PT (**b**); TEM (**c**) and HRTEM (**d**) images of 10% Ag/PT; (**e**–**j**) Elemental mapping images of 10% Ag/PT sample: (**f**) Ag; (**g**) P; (**h**) W; (**i**) Ti; (**j**) O.

The microstructure and morphology of PT nanofibers are presented in Figure 1a. The surface of the nanofibers after calcination at 550 °C is relatively rough and porous, and the fiber diameter is about 80 ± 20 nm. Figure 1b,c show the SEM and TEM images for 10% Ag/PT, respectively. Distinctly, these Ag NPs are equally deposited on the surface of PT with an average diameter of 10 ± 5 nm. The HRTEM images of 10% Ag/PT verify the latticed coexistence of TiO_2_ and Ag in these samples (Figure 1d). The observed lattice spacing of 0.233 nm corresponds to the (112) crystal plane of the anatase phase TiO_2_ (JCPDS no. 21-1272), and the lattice spacing of 0.145 nm corresponds to the Ag (220) plane (JCPDS no. 04-0783). As shown in Figure 1e–j, the elemental mapping images of 10% Ag/PT and the EDS data (Figure S1) further indicated the uniform distribution of Ag, P, W, Ti, and O elements in the sample.

**Figure 2:**
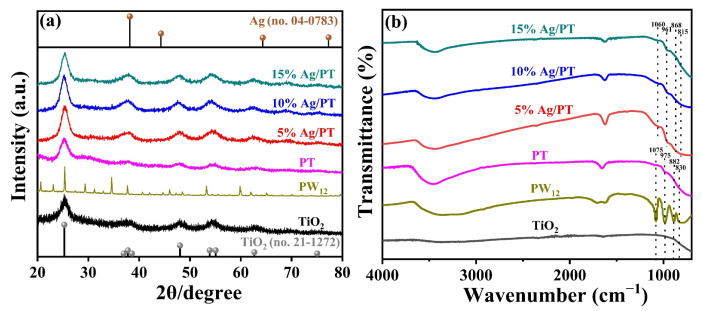
(**a**) XRD and (**b**) FT-IR of the constructed specimens.

The phase composition and purity of the prepared catalysts were investigated with XRD (Figure 2a). For TiO_2_, these characteristic diffraction peaks at 25.3°, 36.9°, 37.8°, 38.5°, 48.0°, 53.9°, 55.0°, and 62.7° are attributed to the (101), (103), (004), (112), (200), (105), (211), and (204) crystal plane of anatase phase TiO_2_ (JCPDS no. 21-1272), respectively [27,28]. With the introduction of PW_12_ into TiO_2_, no peaks of PW_12_ are found in the diffraction peaks of PT, demonstrating the doping of PW_12_ in TiO_2_. When Ag NPs are deposited on PT, the main diffraction peaks of Ag/PT composite are similar to those of PT. Additionally, the main diffraction peak at 38.1°, belonging to Ag (111) phase (JCPDS no. 04-0783), is not obviously found, which might be attributed to the cover effect with diffraction peak of PT [29]. The obtained results certify the presence of PT and Ag NPs in these Ag/PT composites.

Figure 2b displays the FT-IR spectra of various samples. TiO_2_ has no obvious characteristic vibration peak, and the PW_12_ exhibits four characteristic infrared absorption peaks in 700~1100 cm^−1^, including the peaks at 1075, 975, 882, and 830 cm^−1^, respectively. Concretely, the peak at 1075 cm^−1^ is caused by the vibration of the P-O bond, the peak at 975 cm^−1^ is assigned to the vibration of the W=O bond, and the two peaks at 882 and 830 cm^−1^ are attributable to the vibration of the two kinds of W-O_c/e_-W bridge bonds [30,31]. Besides, the peak of PW_12_ near 1600 cm^−1^ may belong to the adsorbed H_2_O molecules [32]. These peaks can be also observed in the PT and Ag/PT materials, indicating the integrity of the PW_12_ Keggin unit in these composites. However, a shift in the vibrational frequencies (1060, 961, 868, and 815 cm^−1^) is detected for Ag/PT, manifesting the presence of interaction between PT and Ag [19]. The aforementioned results certify that the Ag/PT materials have been fabricated successfully.

**Figure 3:**
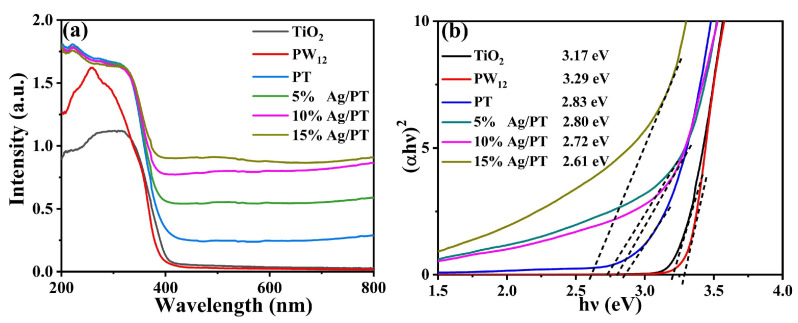
(**a**) UV-Vis absorption spectra and (**b**) the corresponding Tauc plots of obtained specimens.

A UV-Vis diffuse reflectance spectra (DRS) measurement was performed to evaluate the light absorption properties of the obtained specimens. According to Figure 3a, the light absorption edge of TiO_2_, PW_12_ catalysts appeared around 400 and 380 nm. For PT photocatalysts, the light absorption intensity was increased due to the adulteration of PW_12_. In particular, the strongest optical absorption ability in the Ag/PT composites can be attributed to the introduction of Ag NPs [33], which would be beneficial to produce more photogenerated charge carriers to participate in the reaction [34]. We found that the SPR absorption band of Ag NPs ranges from 480 nm to 550 nm (Figure S2) [35]. Furthermore, as shown in Figure 3b, the band gaps of various catalysts were calculated by the following equation: αhν = A(hν−E_g_)^1/2^, in which A, hν, and α represent the constant, photon energy, and absorption coefficient, respectively [36]. 

The band gap values were 3.17, 3.29, 2.83, 2.80, 2.72, and 2.61 eV for TiO_2_, PW_12_, PT, and x% Ag/PT (x = 5, 10 and 15), respectively. The doping of H_3_PW_12_O_40_ introduces additional electronic states and energy levels into the band structure of TiO_2_. These additional electronic states can interact with the electron energy levels of TiO_2_, leading to adjustments in the band structure, thereby reducing the band gap [11,27]. Obviously, in comparison with PT, the band gap of Ag/PT was reduced, which suggests that Ag might introduce a local energy level to the band gap of PT, resulting in a reduced energy gap [37].

**Figure 4:**
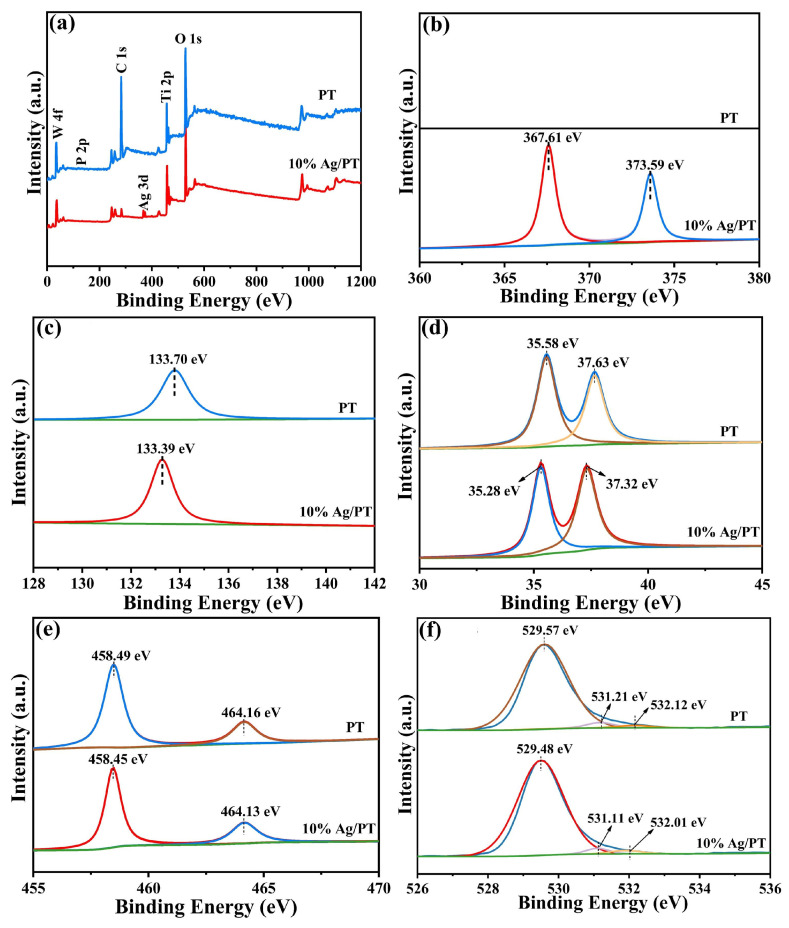
The XPS profiles: (**a**) full spectra; (**b**) Ag 3d; (**c**) P 2p; (**d**) W 4f; (**e**) Ti 2p; (**f**) O 1s.

The composition and chemical state information of as-prepared specimens were probed with X-ray photoelectron spectroscopy (XPS). The elemental composition of 10% Ag/PT was demonstrated by the signal detection of P, W, O, Ti, and Ag elements in the full XPS spectra (Figure 4a). Figure 4b–f shows the high resolution XPS profiles for Ag 3d, P 2p, W 4f, Ti 2p, and O 1s of PT and 10% Ag/PT, confirming the successful preparation of the composites. As presented in Figure 4b, the 10% Ag/PT composite showed two peaks at Ag 3d, located at 367.61 eV and 373.59 eV, belonging to Ag^0^ 3d_5/2_ and Ag^0^ 3d_3/2_ metallic silver monomers, respectively [38,39]. The P 2p XPS profile for PT (Figure 4c) has a peak at 133.70 eV, and this binding energy was considered to be the presence of P^5+^ [40]. The P 2p peak of 10% Ag/PT was shifted towards the lower binding energy region in comparison with PT. In the PT material, the high-resolution XPS spectrum of the W 4f region (Figure 4d) showed two peaks at 35.58 eV and 37.63 eV for the W 4f_7/2_ and W 4f_5/2_ binding energies, respectively, and, in 10% Ag/PT, W 4f was shifted toward the lower binding energy with binding energies of 35.28 eV and 37.32 eV [41,42]. Figure 4e shows the presence of Ti 2p_3/2_ and Ti 2p_1/2_ characteristic peaks observed at 458.49 eV and 464.16 eV in PT, which are features of Ti^4+^ in TiO_2_ [43]. Notably, the binding energies of Ti 2p XPS for 10% Ag/PT were shifted to 458.45 eV and 464.13 eV, providing evidence of the interaction between PT and Ag [44]. Figure 4f shows the XPS spectra of O 1s. Two peaks, at 529.57 eV (PT) and 529.48 eV (10% Ag/PT), were found, which were considered as Ti-O [45]; meanwhile, two peaks are found at 531.21 eV and 532.12 eV (PT) and 531.11 eV and 532.01 eV (10% Ag/PT), corresponding to W-O and P-O, respectively [46]. Notably, these peaks in 10% Ag/PT composites shifted to lower binding energies compared to PT, which indicated the presence of interfacial interaction between Ag and PT [47].

**Figure 5:**
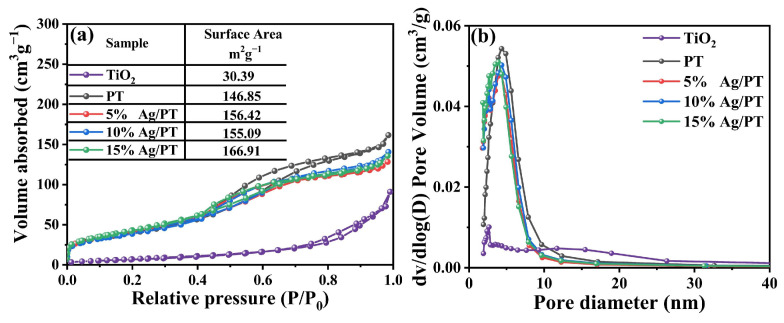
(**a**) N_2_ adsorption-desorption isotherms and (**b**) pore size distributions of as-synthesized samples.

Figure 5a demonstrates that the N_2_ adsorption and desorption isotherms of different specimens conform to type IV, while the hysteresis line follows type H1, indicating the presence of a mesoporous structure [48,49]. The specific surface areas (SSA) were 30.39, 146.85, 156.42, 155.09, and 166.91 m^2^/g for TiO_2_, PT and x% Ag/PT (x = 5, 10 and 15), respectively. The result suggested that the introduce of PW_12_ is beneficial to enhance the SSA of TiO_2_, which would demonstrate an improved catalytic performance. Figure 5b presents the pore size distributions of as-obtained samples. The average pore volumes were 11.57, 5.32, 4.25, 4.61, and 4.40 nm for TiO_2_, PT, and x% Ag/PT (x = 5, 10 and 15), respectively. It is clear that the average pore volume of Ag/PT composites decreased, which might be due to the accumulation of Ag NPs on the PT surface.

### 2.2. Catalytic Activity Assessment of Ag/PT Composites

#### 2.2.1. Photocatalytic Removal of TC

**Figure 6:**
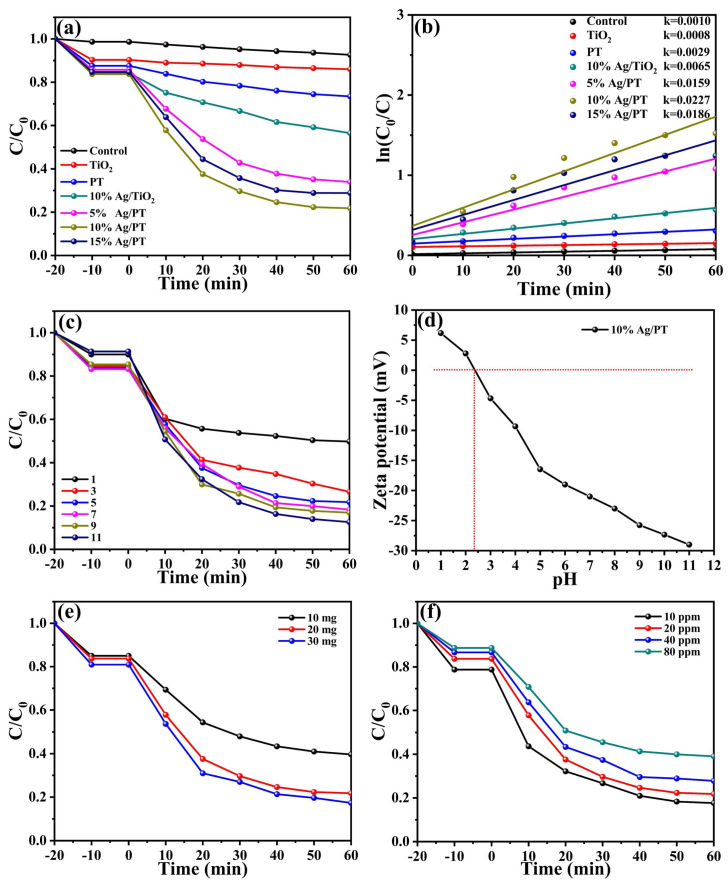
(**a**) The visible-light (λ > 420 nm) degradation of TC utilizing various specimens (catalyst usage: 20 mg; TC: 20 mL of 20 ppm; pH = 7); (**b**) The pseudo-first-order kinetic study for TC degradation; Degradation of TC with 10% Ag/PT with various conditions: (**c**) Different pH values (TC: 20 mL of 20 ppm; catalyst usage: 20 mg); (**d**) Zeta potential of 10% Ag/PT at different pH values; (**e**) Diverse catalyst amount (TC: 20 mL of 20 ppm; pH = 7); (**f**) Different concentration of TC (TC: 20 mL; pH = 7; catalyst amount: 20 mg). Light source: 300 W Xe light (CEL-HXF300, AULIGHT).

TC was chosen as an organic pollutant to explore the photocatalytic capacity of obtained samples [50,51]. As presented in Figure 6a, the adsorption-desorption equilibrium was reached between the catalyst and TC under dark conditions within 20 min. The control experiment was designed and demonstrated that the self-photolysis process of TC can be excluded. TiO_2_ exhibits a negative effect on the TC degradation. The degradation efficiencies of TC on PT and 10% Ag/TiO_2_ were significantly higher compared to pure TiO_2_, which reached 26.53% and 43.52% within 60 min, respectively. This indicates that the photocatalytic activity of TiO_2_ can be improved with the proper introduction of H_3_PW_12_O_40_ or Ag NPs. Moreover, the photocatalytic property of Ag/PT was further boosted, benefiting from the remarkable contribution of the SPR effect originating from the Ag NPs. The 10% Ag/PT composite shows the optimal degradation efficiency of 78.19% (Figure S3a), which exhibits better performance compared to numerous other catalysts, in terms of TC removal (Table S1). Besides, the removal of total organic carbon (TOC) for TC degradation reached 60.08% within 1 h using 10% Ag/PT material (Figure S4), which implies that the TC degradation was incomplete. Nevertheless, when more Ag was deposited on the PT, the TC removal rate of the synthesized 15% Ag/PT composite reduced to 71.12%. Because excessive Ag occupies a part of the active sites of PT, the adsorption capacity and degradation rate of Ag/PT composite towards TC molecules is reduced.

As presented in Figure 6b, the fitting results of the TC degradation rate indicate that it was in accordance with the first-order kinetic model. Distinguishingly, the reaction rate constant k for TC degradation with 10% Ag/PT was 0.0227 min^−1^, which was about 29- and 8-times higher than those of TiO_2_ and PT, respectively. Therefore, the doping of PW_12_ and the modification of Ag NPs are effective methods to boost the photocatalytic performance of TiO_2_.

Effect of different pH values: The degradation of TC in aqueous solution undergoes protonation and deprotonation reactions, and the pH of the solution will lead to different charge states, which affects the decomposition of TC. As shown in Figure 6c, the TC degradation efficiency gradually increased with the increase of pH, which achieved the optimal value of 87.42% at pH 11. The alkaline environment favors the generation of •O_2_^−^, which is one kind of active species during the pollutant degradation process [52]. Besides, TC molecules exhibit a high susceptibility to photolysis in alkaline conditions, benefiting from the transition from the π to π* states of the (HOMO-1 to LUMO) chromophore [53]. At neutral pH, the TC removal rate was 78.19% after 60 min of light exposure. However, under acidic conditions, the degradation efficiency of TC further decreased. In Figure 6c, the adsorption removal efficiency of TC by 10% Ag/PT at different pH conditions were 10.04% (pH 1.0), 15.41% (pH 3.0), 16.28% (pH 5.0), 16.78% (pH 7.0), 14.61% (pH 9.0), and 8.67% (pH 11.0). This may be related to the zeta potential of the catalyst, which was examined for 10% Ag/PT at different pH conditions (Figure 6d). Obviously, the zeta potential of 10% Ag/PT was positive at pH < 2.4 and negative at pH > 2.4. Moreover, when pH < 3.3, TC appeared as a cation (TCH_3_^+^); when pH = 3.3~7.7, TC existed as an ampholyte (TCH_2_^0^); when pH was greater than 7.7, TC appeared as an anion (TCH_3_^−^) [54]. Therefore, when pH = 1.0, the surface of 10% Ag/PT was positively charged and the TC molecules were present in the protonated (TCH_3_^+^, pH < 3.3), which generated an intense electrostatic repulsion and weak adsorption ability. With the increase of pH from 3 to 7, the positive surface charge of 10% Ag/PT decreased from −4.64 mV to −21.07 mV, and the TC molecules were in neutral (TCH_2_^0^, pH 3.3–7.7), indicating that the electrostatic repulsion was suppressed, thus promoting the adsorption capacity. When the pH was 9.0 and 11.0, the electrostatic repulsion existed between the catalyst with a negative charge and TC (TCH_3_^−^, pH > 7.7). Furthermore, the excess OH^−^ could occupy the adsorption sites of the catalyst, generating a slight reduction of adsorption ability [55].

Influence of catalyst dosage: As shown in Figure 6e, the degradation efficiency was significantly enhanced from 60.35% to 78.19%, with the catalyst quantity from 10 to 20 mg, which could be assigned to the increase of active sites [56]. However, the TC degradation rate increased indistinctively (78.19% to 82.64%) upon further increasing the catalyst usage from 20 to 30 mg, which may be due to the poor light transmission of the solution applying too much catalyst [57].

Effects of initial TC concentration: Figure 6f provides the effect of TC concentration on the photodegradation performance. The TC degradation rate decreased continuously, with the TC concentration ranging from 10 to 80 ppm. The explanation may be that the limited number of photogenerated carriers lead to restrict TC degradation when the initial TC concentration was too high. In addition, the higher TC concentration affected the penetration ability of photons and, thus, negatively affects the photocatalytic activity [58].

#### 2.2.2. Photocatalytic Degradation of ENR and MO

**Figure 7:**
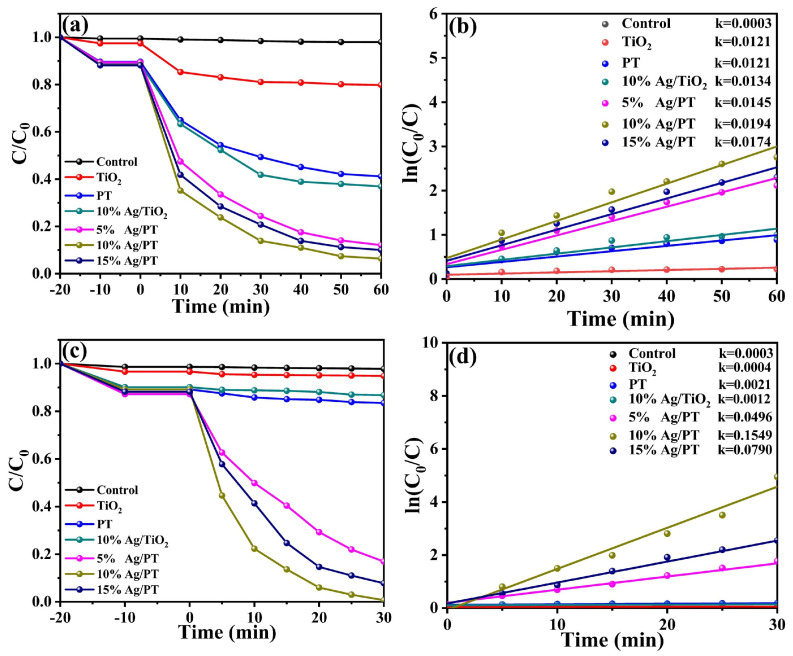
(**a**) Visible-light removal curses of ENR by different specimens; (**b**) Reaction rate constant k; The degradation profiles (**c**) and reaction rate constant k (**d**) of MO degradation.

The catalytic performance for Ag/PT composites were further evaluated by degrading ENR and MO in visible-light. During the dark reaction, the pollutants molecules were adsorbed on the photocatalyst surface for 20 min to obtain the adsorption-desorption equilibrium. As presented in Figure 7a, the photocatalytic degradation efficiencies of ENR with control, TiO_2_, 10% Ag/TiO_2_, PT, 5% Ag/PT, 10% Ag/PT, and 15% Ag/PT were 1.99%, 20.17%, 58.84%, 63.09%, 87.93%, 93.65%, and 89.98%. Specially, 10% Ag/PT had the best photocatalytic activity of 93.65% (k = 0.0194) (Figure 7b and Figure S3b), which was 4.64-, 1.48-, and 1.59-times higher than that of TiO_2_, 10% Ag/TiO_2_, and PT, respectively. Similarly, the degradation profiles in Figure 7c manifesting 10% Ag/PT also displayed an excellent MO degradation rate of 99.29% (k = 0.1549) (Figure 7d and Figure S3c). The influencing parameters of catalyst dosage and MO concentration were also studied in Figure S6. Moreover, the degradation efficiencies of Ag/PT composites are superior to other catalysts for ENR and MO removal (Tables S2 and S3). These data verify that as-prepared Ag/PT is one kind of multi-functional material in the field of environmental remediation. 

### 2.3. Stability Test of Photocatalyst

**Figure 8:**
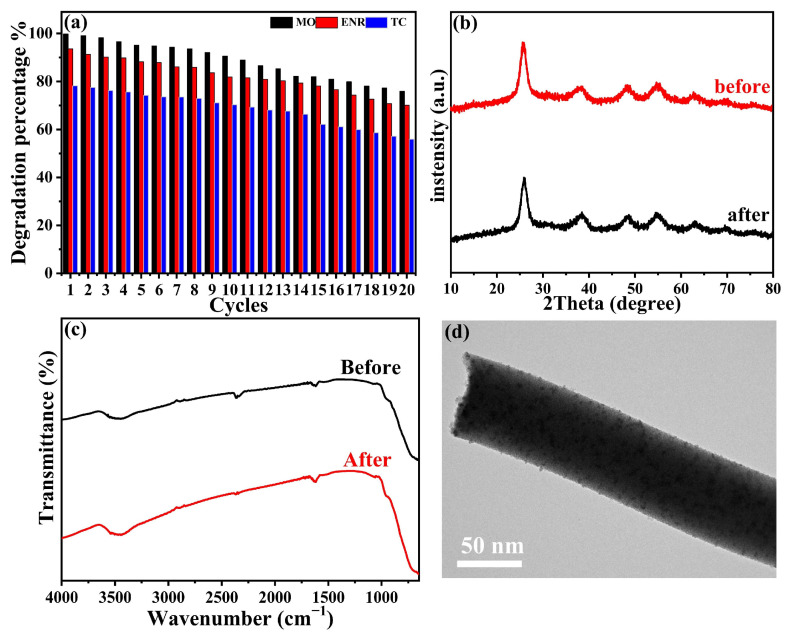
(**a**) The cyclic experiments for removing TC, ENR, and MO by 10% Ag/PT; XRD (**b**), FT-IR (**c**) TEM image (**d**) for 10% Ag/PT before and after use in TC degradation.

Figure 8a shows the cycling experiments of 10% Ag/PT as a visible-light catalyst for the degradation of various contaminants. After 20 cycles of reuse, the degradation efficiency of MO, ENR, and TC exhibited a slight decrease, and by using ICP-6000 test, the leaching amount of Ag after degradation was 2.1 ppm, indicating that the as-obtained Ag/PT composites had good reuse performance. Moreover, the photocatalytic stability of Ag/PT materials was confirmed with XRD and FT-IR. As shown in Figure 8b,c, the XRD diffraction peaks and FT-IR spectra of the used 10% Ag/PT remained unchanged in comparison with the fresh sample, verifying the good structural stability of these materials. Furthermore, the TEM image after TC removal (Figure 8d) also demonstrated the good cycling stability of the catalyst.

### 2.4. Photocatalytic Mechanism Investigation

#### 2.4.1. Photogenerated Carriers Behavior Analysis

**Figure 9:**
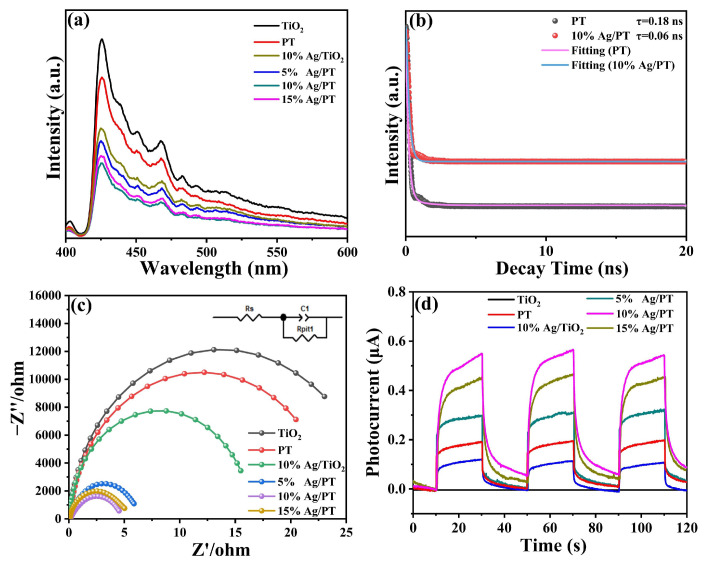
The steady PL (**a**), transient PL (**b**), EIS (Insert: impedance equivalent circuit diagram) (**c**) and photocurrent (**d**) of various samples.

The photoluminescence (PL) spectra were measured to reflect the separation efficiency of photoinduced carriers from the synthesized catalysts. As demonstrated in Figure 9a, these materials exhibited similar peaks at 425 nm. The fluorescence intensity for Ag/PT composite exhibited a significant decrease compared to TiO_2_, PT, and 10% Ag/TiO_2_, implying that the recombination of photogenerated charge carriers was effectively suppressed [59,60]. In addition, the 10% Ag/PT catalyst had the lowest peak intensity, implying a higher separation rate of electron-hole pairs and better catalytic capacity compared to the remaining specimens. The fluorescence lifetimes of PT and 10% Ag/PT were determined by time-resolved fluorescence attenuation spectrometry (TRPL). As revealed in Figure 9b, the fluorescence intensity of PT and 10% Ag/PT both decreased exponentially. The average fluorescence lifetime τ_ave_ of PT and 10% Ag/PT were calculated to be 0.18 ns and 0.06 ns, respectively (Table S4). The result shows that 10% Ag/PT has a shorter average decay time than PT, which indicates that the deposition of Ag nanoparticles is beneficial to delay the recombination of photoinduced carriers [61]. The corresponding quenching and lifetime reduction of TRPL implies a high non-radiative decay rate at 10% Ag/PT, and the establishment of a fast electron transfer pathway for accumulated photoproduced electrons is conducive to the enhancement of catalytic capacity [62].

The electrochemical impedance spectroscopy (EIS) and instantaneous photocurrent have been employed for examining the separation and migration ability of photogenerated electron-hole pairs. Figure 9c illustrates the EIS Nyquist plots form distinct electrodes, and the equivalent circuit are provided as an insert. Generally, the small EIS radian of the electrochemical impedance corresponds to the low charge transfer resist [63]. It is clear that the radius of these Ag/PT materials were much smaller than those of TiO_2_, PT, and 10% Ag/TiO_2_. Specially, 10% Ag/PT has the smallest radius, which strongly manifested that the composite possessed fastest transfer and migration ability of carriers [64]. Additionally, Figure S7 presents the Bode plots of PT and 10% Ag/PT, which confirmed a prolonged lifetime of photoinduced electrons for 10% Ag/PT in comparison to PT. The photocurrents of obtained specimens were measured in Figure 9d. The photocurrent was found to be stable and reproducible in three cycles. The photocurrent density obeyed the following order: 10% Ag/PT > 15% Ag/PT > 5% Ag/PT > 10% Ag/TiO_2_ > PT > TiO_2_. Specifically, the photocurrent density of 10% Ag/PT (0.23 μA/cm^2^) was much larger than that of PT (0.09 μA/cm^2^) and 10% Ag/TiO_2_ (0.05 μA/cm^2^), which would lead to a remarkable enhancement in photocatalytic capability [65]. The results of various measurements collectively demonstrated that the Ag/PT composites have low charge transfer resistance and high separation efficiency of photogenerated carriers, which would reveal an outstanding catalytic performance.

#### 2.4.2. Active Species in Photocatalytic Reactions

**Figure 10:**
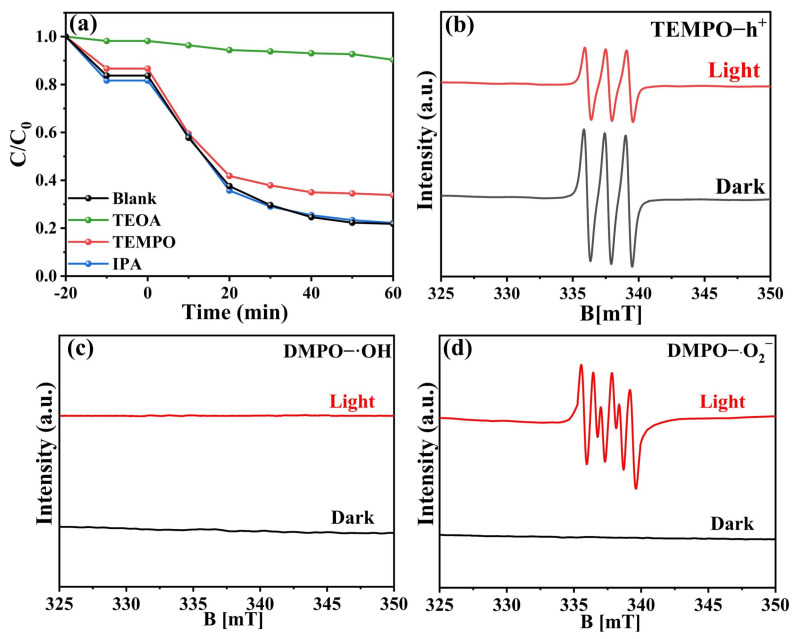
(**a**) The degradation of TC with diverse scavenges by 10% Ag/PT sample; (**b**) ESR spectra of TEMPO-h^+^; ESR signals of (**c**) DMPO-·OH and (**d**) DMPO-·O_2_^−^.

To elucidate the degradation mechanism of TC, the radical capture experiments were performed, and the results were presented in Figure 10a. Herein, 4-hydroxymethylpropane (TEMPO, ·O_2_^−^ quencher), triethanolamine (TEOA, h^+^ quencher), and isopropyl alcohol (IPA, ·OH quencher) were employed as free radical trapping agents [66,67]. Distinctly, the addition of TEOA to the reaction system significantly inhibited the degradation efficiency, and the addition of TEMPO also reduced the degradation activity to some extent, verifying the important function of h^+^ and ·O_2_^−^ in TC degradation. Meanwhile, the degradation rate was almost unchanged with the addition of IPA, implying that ·OH was not the dominating active substance.

To directly verify the reactive species involved in the reaction process, electron spin resonance (ESR) measurement was conducted, applying 5,5-dimethyl-1-pyrroline N-oxide (DMPO) and 2,2,6,6-Tetramethyl-1-piperidinyloxy (TEMPO) as spin-trapping agents [68]. TEMPO can trap the photogenerated holes and form TEMPO-h^+^ spin-products, which exhibit silent ESR signals. As displayed in Figure 10b, under dark conditions, three distinctive peaks corresponding to the TEMPO were identified, which were obviously declined under visible-light, demonstrating the production of TEMPO-h^+^ spin-products [69]. Meanwhile, ·OH and ·O_2_^−^ can be captured with DMPO, generating evident ESR signals. In Figure 10c, no characteristic peaks were found under both dark and light conditions in the ·OH test, indicating that ·OH did not play a role in the catalytic reaction. In Figure 10d, in the ·O_2_^−^ test, no characteristic peaks were detected under dark conditions; nevertheless, the characteristic peaks corresponding to DMPO-·O_2_^−^ were clearly observed upon visible-light irradiation, authenticating successful generation of ·O_2_^−^ radicals. These results indicated that the photodegradation of TC was primarily driven with the involvement of ·O_2_^−^ radicals and h^+^.

#### 2.4.3. Degradation Pathways of TC and Toxicity Assessment

**Figure 11:**
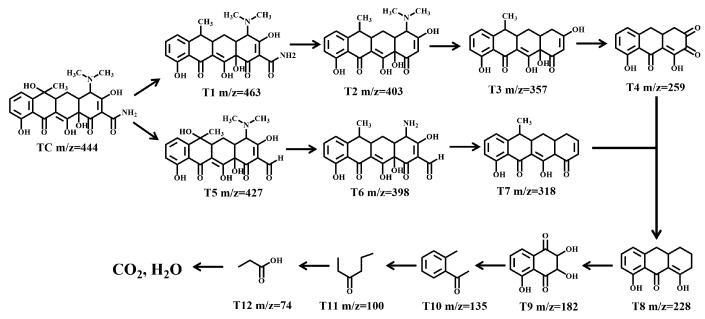
The probable catalytic degradation pathways for TC.

As revealed in Figure 11 and Figure S5, the pathways of TC photodegradation were explored by HPLC-MS. The molecular weight of TC is expressed as the product *m*/*z* = 444. Figure 11 summarizes and illustrates two possible degradation pathways. In pathway 1, the intermediate of T1 (*m*/*z* = 463) may be derived from the dehydroxylation of TC, after which T1 forms T2 (*m*/*z* = 403) through the deamidation process. Intermediate with T3 (*m*/*z* = 357) is resulted from loss of one N-2 methyl group. The product T4 (*m*/*z* = 259) is obtained by the ring-opening reaction of T3. Pathway 2 is the transition from TC to T5 (*m*/*z* = 427) after deamination. Then, T5 is dehydroxylated and dedimethylated to T6 (*m*/*z* = 398), which is deaminated and demethylated to T7 (*m*/*z* = 318). After T4, T8 is formed by the break of double-bond oxygen, and T7 is formed by ring-opening and dehydroxylation. After continuous ring-opening reactions, T8 forms T9 (*m*/*z* = 228), T10 (*m*/*z* = 182), T11 (*m*/*z* = 100), and T12 (*m*/*z* = 74). Further degradation of intermediates can produce small molecules such as CO_2_, H_2_O, and inorganic ions. According to the above analysis, it can be inferred that photocatalytic degradation of tetracycline involves deamidation, dehydroxylation, and ring-opening reactions [3,70]. 

**Figure 12:**
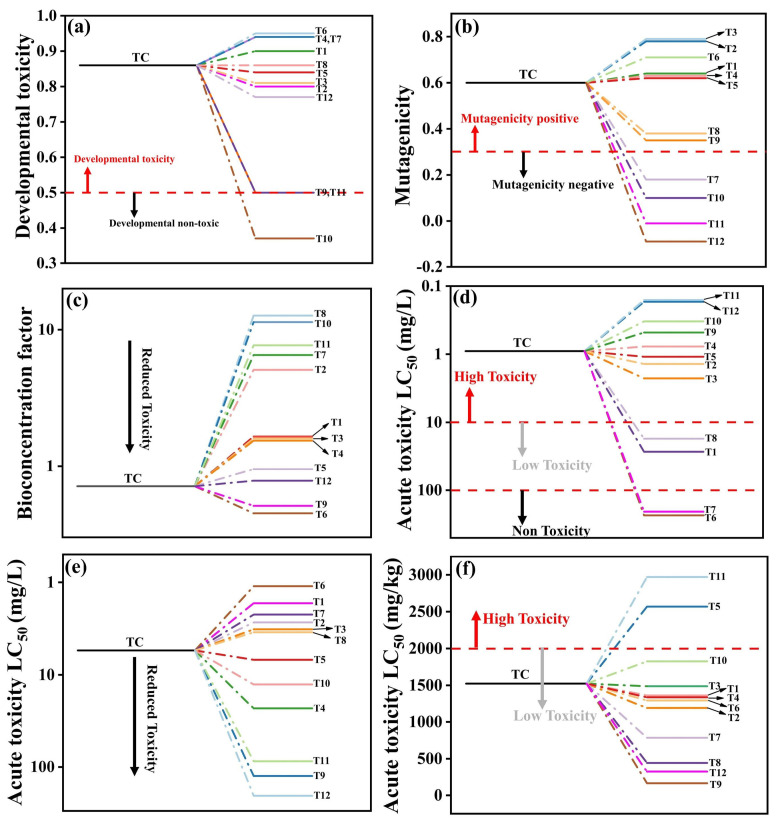
(**a**) Developmental toxicity; (**b**) mutagenicity; (**c**) bioconcentration factor; (**d**) fathead minnow, (**e**) Daphnia magna (**f**), and oral rat for these products in TC degradation.

Furthermore, we investigated the toxicity of TC and its 12 intermediates using QSAR prediction with a toxicity estimation software tool (T.E.S.T. Version 5.1.2) [71]. Figure 12a,b show that TC was “developmentally toxic” and “mutagenic positive” [72]. One developmentally non-toxic TC intermediate (T10) and four mutagenic-negative TC intermediates (T7, T10, T11, T12) were produced after light treatment. Furthermore, most intermediates were less toxic than TC. As illustrated in Figure 12c, the bioaccumulation factors of intermediates T9 and T6 were lower than those of TC, and the photodegradation process could reduce the bioaccumulation factor for TC, which was primarily attributed to the hydroxylation reaction [73].

In Figure 12d–f, three evaluation indicators were used to evaluate the acute toxicity of TC and its intermediates: (i) Fathead minnow LC50 (96 h) represents the concentration at which 50% of fathead minnows are killed after 96 h; (ii) Daphnia magna LC50 (48 h) represents the concentration at which 50% of Daphnia magna are killed after 48 h; and (iii) Oral rats LD50 represents the concentration at which 50% of rats are killed after 48 h of oral ingestion. The LC50 values of 0.90 mg/L for blackhead minnow, 12.70 mg/L for Daphnia magna, and 1105.75 mg/kg for TC in rats were defined as “highly toxic”, “harmful”, and “toxic” compounds, respectively [74]. Obviously, T1, T6, T7, and T8 intermediates all showed low LD50 values (Figure 12d). Daphnia magna showed lower LC50 values than TC intermediates, except for T6, T7, T1, T2, T3, and T8 (Figure 12e). With the exception of intermediates T5 and T11, rats exhibited lower toxicity to TC intermediates (Figure 12f). According to the aforementioned toxicity prediction results, the toxicity of several intermediates still exists, which could be reduced by extending the reaction time.

#### 2.4.4. Possible Photocatalytic Mechanism

**Figure 13:**
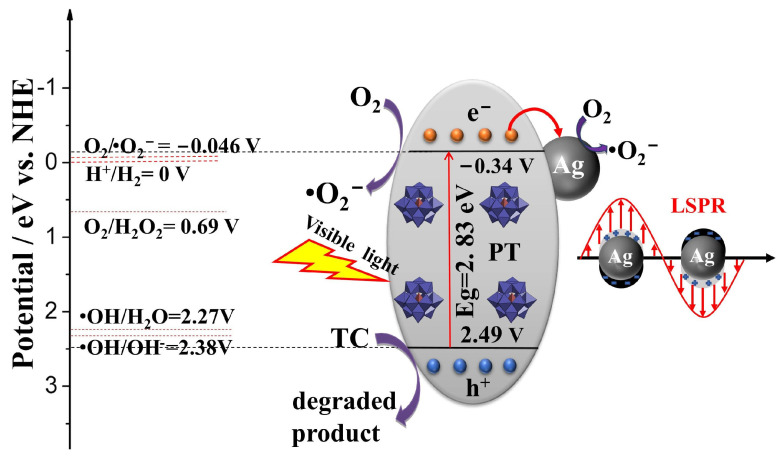
The photocatalytic mechanism of TC degradation using Ag/PT system.

In Figure S9, the tangent slope of the Mott-Schottky profile reflects that PT belongs to n-type semiconductor. The E_fb_ of PT relative to Hg/Hg_2_Cl_2_ was found to be −0.17 eV. Given that the conduction band energy (E_CB_) of n-type semiconductor is approximately 0.2 eV higher than the flat band potential (E_fb_) [75], the E_CB_ for PT could be determined as −0.13 eV (vs. NHE), according to E_NHE_ = E_Hg/Hg2Cl2_ + 0.242 eV. From the (αhv)^2^ vs. hv plot (Figure 3b), the band gap energy (E_g_) of PT is calculated to be 2.83 eV. Therefore, the VB (valence band) edge position of PT (E_VB_ = E_CB_ + E_g_) is determined to be 2.70 eV [76]. Based on the aforementioned results, the catalytic mechanism for TC degradation by Ag/PT system with visible-light was proposed (Figure 13). The PT was photoexcited to generate electrons and holes under visible-light irradiation (Equation (1)). Meanwhile, a large number of hot electrons are produced, due to the surface plasmon resonance (SPR) effect of Ag NPs [77,78]. The Ag NPs serving as electron traps could effectively capture photoinduced electrons on the CB of PT, while the Schottky barrier established by Ag^0^ could promote the transfer of SPR-excited electrons, further accelerating the charge separation (Equation (2)). These electrons on Ag NPs react with O_2_ to form ·O_2_^−^ participating in oxidation reaction (Equations (3) and (4)). Moreover, the photoinduced holes in PT directly oxidize TC according to the result of ESR measurements and capturing tests (Equation (5)). Ultimately, TC was efficiently removed with the help of h^+^ and ·O_2_^−^ active species (Equation (6)).

Ag/PT + hν→Ag/PT (h^+^ + e^−^) (1)

Ag/PT (h^+^ + e^−^)→PT (h^+^) + Ag (e^−^) (2)

O_2_ + Ag (e^−^)→•O_2_^−^ + Ag (3)

•O_2_^−^ + TC→CO_2_ + H_2_O (4)

PT (h^+^) + TC→PT + H_2_O + CO_2_ (5)

h^+^/•O_2_^−^ + TC→intermediate products→CO_2_ + H_2_O (6)

## 3. Experiments and Characterizations

### Construction of Ag/PT Photocatalysts

**Scheme 1:**
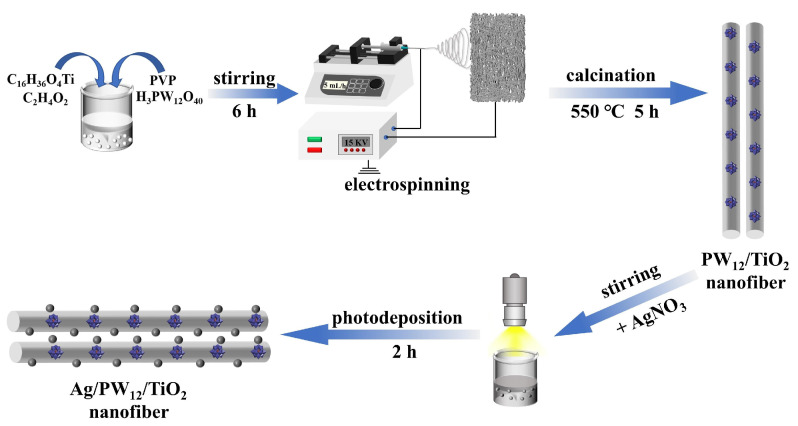
Schematic diagram for the fabrication process of Ag/PT composite.

As shown in Scheme 1, Ag/PT composite nanofibers were prepared employing electrospinning/photoreduction methods. First of all, PT nanofibers were synthesized by the electrospinning/calcination method. Briefly, PVP was dissolved in a mixture of anhydrous ethanol, acetic acid, and tetrabutyl titanate, and stirred for 1 h. PW_12_ was then added and stirred until complete dissolution. The homogeneous precursor solution was subjected to electrostatic spinning operation, followed by calcination, to prepare PT nanofibers. Secondly, Ag NPs were modified on the PT nanofibers by photoreduction. PT nanofibers powder was added to the solution of V_water_:V_isoprobanol_ = 1:1, which was then sonicated for 30 min. Then, the solution was evacuated, and the suspension was illuminated for 1 h using a 300 W xenon lamp with full spectrum light. Then, AgNO_3_ solution was added and stirred for 60 min. The Ag/PT composite was prepared.

The fabrication and characterization methods of Ag/PT composites are displayed in the Supplementary Material.

## 4. Conclusions

Herein, a novel Ag/PT composite material has been constructed utilizing electrospinning/photoreduction methods, which exhibited remarkable photocatalytic activities for degradation TC, ENR, and MO. The results of mechanism investigation showed that the excellent catalytic property could be due to the following two reasons: (1) the doping of PW_12_ to TiO_2_ can enhance the utilization of visible spectrum and redox reaction activity of titanium dioxide; (2) the precious metal Ag possesses the LSPR effect, which can improve the utilization of sunlight and generate more charge carriers. Besides, the LSPR effect will have a high-intensity small range electromagnetic field, which will greatly improve the separation rate of photogenerated electron-hole pairs. Moreover, the degradation intermediates and pathways were revealed through HPLC-MS. The toxicity of TC degradation products was also investigated using QSAR prediction. This current work offers novel thoughts for developing efficient and stable catalysts for environmental remediation.

## Data Availability

Data are contained within the article and Supplementary Materials.

## Supplementary Materials

The following supporting information can be downloaded at: https://www.mdpi.com/article/10.3390/molecules28196831/s1, Figure S1: EDX data of 10% Ag/PT sample; Figure S2: UV-Vis absorption spectra of 5%, 10% and 15% Ag/PT sample; Figure S3: The profiles of photocatalytic degradation of TC (a), ENR (b) and MO (c) by 10% Ag/PT under visible-light irradiation (λ > 420 nm); Figure S4: The TOC removal (%) for TC degradation by 10% Ag/PT sample; Figure S5: Photodegradation of TC with 10% Ag/PT under Diverse water quality (catalyst amount: 20 mg; TC: 20 mL of 20 ppm; pH = 7). Figure S6: Degradation of MO with 10% Ag/PT with various conditions: (a) Diverse catalyst amount (MO: 20 mL of 20 ppm; pH = 1) and (b) Different concentration of MO (MO: 20 mL; pH = 1; catalyst amount: 20 mg). Figure S7: The Bode plots of PT and 10% Ag/PT composite; Figure S8: The main intermediate products generated during the photocatalytic TC degradation process: (a) 0 min; (b) 30 min; (c) 60 min with 10% Ag/PT as catalyst; Figure S9: The E_fb_ of PT (V vs. Hg/Hg_2_Cl_2_). Table S1: The comparison of TC degradation activity of 10% Ag/PT with previous literatures; Table S2: The comparison of ENR degradation activity of 10% Ag/PT with previous literatures; Table S3: The comparison of MO degradation activity of 10% Ag/PT with previous literatures; Table S4: Fitted parameters of the TRPL decay profiles. References [79,80,81,82,83,84,85,86,87,88,89,90,91,92,93,94,95,96,97,98,99,100,101,102,103,104,105,106,107,108,109,110,111,112,113,114,115,116,117,118,119,120,121,122] are cited in the Supplementary Materials.

## Sample Availability

Samples of the compounds are available from the authors.
