# Supplementary material for: Boosted Photocatalytic Performance for Antibiotics Removal with Ag/PW12/TiO2 Composite: Degradation Pathways and Toxicity Assessment
Source: Molecules. 2023 Sep 27;28(19):6831. doi: 10.3390/molecules28196831 (PMC10574183; doi:10.3390/molecules28196831)
Supplement: Supplementary file 1 [file molecules-28-06831-s001.zip › molecules-2626100-supplementary.pdf]

Supplement Files

Boosted Photocatalytic Performance for Antibiotics Removal with Ag/PW_{12}/TiO_2 Composite: Degradation Pathways and Toxicity Assessment

Hongfei Shi [1,*], Haoshen Wang [1], Enji Zhang [1], Xiaoshu Qu [1], Jianping Li [1,*], Sisi Zhao [2], Huajing Gao [1] and Zhe Chen [1]

1. Institute of Petrochemical Technology, Jilin Institute of Chemical Technology, Jilin, 132022, China;
2. Institute of Catalysis for Energy and Environment, College of Chemistry & Chemical Engineering, Shenyang Normal University, Shenyang, Liaoning 110034, China

*. Correspondence: shihf813@nenu.edu.cn (H.S.); lijp156@nenu.edu.cn (J.L.)

S1. Experimental sections

S1.1. Chemicals and Reagents

Phosphotungstic acid ($H_3PW_{12}O_{40}{\cdot}xH_2O$, AR), Titanium butoxide ($C_{16}H_{36}O_4Ti$, AR), Acetic acid ($C_2H_4O_2$, ≥ 99.5%, AR), Polyvinylpyrrolidone (PVP, Mw = 1300000, AR) Ethylene glycol ($C_2H_6O_2$, ≥98.0%, AR), Sodium sulfate (Na_2SO_4, ≥99.0%, AR)，Triethanolamine (TEOA, 98%, AR), Isopropyl alcohol (IPA, ≥99.5%, AR), 4-hydroxy-TEMPO ($C_6H_{15}NO_3$, AR), 5,5-Dimethyl-1-pyrroline N-oxide (DMPO, 97%, AR), Tetracycline (TC, AR), Methyl orange (MO, AR), Enrofloxacin ($C_{19}H_{22}FN_3O_3$, ≥98% AR) and Sodium hydroxide (NaOH, 95%, AR) were obtained from Macklin Biochemical Technology Co., Ltd., China. Potassium ferricyanide ($K_3Fe(CN)_6$, ≥99.5%, AR), Potassium ferrocyanide trihydrate ($K_4Fe(CN)_6{\cdot}3H_2O$, 99%, AR), Barium sulfate ($BaSO_4$, 99%, AR) and Silver nitrate ($AgNO_3$, >99.8%, AR) were purchased from Aladdin Reagent Co., Ltd., China. Hydrochloric acid (36%～38%, AR) and Anhydrous ethanol were purchased from Beijing Chemical works. They were used as received without further purification. Deionized water was used throughout the work.

S1.2. The Preparation of composite catalysts

Preparation of PW_{12}/TiO_2 material

PW_{12}/TiO_2 nanofibers (NFs) were synthesized by an electrospinning/calcination technique. 0.7 g of Polyvinyl Pyrrolidone (PVP, Mw ≈ 1,300,000) was dissolved in a mixture of anhydrous ethanol (15 mL), acetic acid (0.2 mL) and tetrabutyl titanate (TBT, 0.4 mL) with stirring for 1 hour, then 0.072 g of $H_3PW_{12}O_{40}$ (dried at 80℃ for 4 h; 20 mol% relative to TBT) was added, which was stirred until completely dissolved. The homogeneous precursor solution was put into a syringe (20 mL) connected with a 22 gauges blunt needle. The solution feed rate was set as 0.5 mL/h and 15 kV was applied to the needle and collector covered by aluminum foil. The distance of 15 cm was applied between the counter electrode and the syringe tip. The collected nanofibers were calcined at 550 ℃ for 5 h (heating rate: 2 ℃/min) to fabricate the PW_{12}/TiO_2 NFs.

Preparation of $Ag/PW_{12}/TiO_2$ composite

The PW_{12}/TiO_2 nanofibers were decorated with Ag NPs through a photodeposition method. Typically, 200 mg of PW_{12}/TiO_2 powder and designed amount of $AgNO_3$ (20 mM·L^{-1}) solution were added into a 60 mL solution (V_{H2O}:$V_{isopropanol}$=1:1), followed with sonication for 0.5 h. The suspension was subjected to irradiation for 60 min provided by a 300 W Xe lamp with a light intensity of 100 mW/cm^2. These resulting powders were collected, then washed with absolute ethyl alcohol and deionized water repeatedly, finally dried at 80 ^oC over 6 hours. The weight ratio of Ag to PW_{12}/TiO_2 was adjusted by the added amount of $AgNO_3$ solution. Three Ag-decorated PW_{12}/TiO_2

samples with different weight ratios of Ag to PW_{12}/TiO_2 (theoretical Ag loadings (wt%) = 5, 10 and 15) were obtained and are denoted as (5%, 10% and 15%) $Ag/PW_{12}/TiO_2$, respectively.

Preparation of 10% Ag/TiO_2 composite

The preparation method is the same as 10% $Ag/PW_{12}/TiO_2$ without adding $H_3PW_{12}O_{40}$.

S1.3. Characterization Methods

The crystal structures of prepared samples were measured using a Bruker AXS D8 Focus X-ray diffractometer (XRD) with Cu Kα radiation (λ= 1.54056 Å) in the range of 10°-80°. Fourier transform infrared (FTIR) spectra were carried out using an Alpha Centaurt FTIR spectrometer in the frequency range 4000-400 cm^{-1} at room temperature. X-ray photoelectron spectroscopy (XPS) measurement was performed to analyze the chemical state and composition on an ESCALABMKII spectrometer with Al-Kα radiation (hν =1486.6 eV). Scanning electron microscopy (SEM, JEOL JSM 4800F) coupled with an energy-dispersive X-ray (EDX) spectrometer was employed to investigate the morphology of the photocatalysts, and the transmission electron microscopy (TEM) and HRTEM images were taken on a JEM-2100F microscope operated at 200 kV. The Brunauer-Emmett-Teller (BET) specific surface areas were performed on a Micromeritics ASAP-2460 Automatic specific surface area and porous physical adsorption analyzer. The UV-Vis diffuse reflection spectra (DRS) were obtained with a Shimadzu UV-2650 UV-vis spectrophotometer with wavelength range of 200-800 nm with $BaSO_4$ as a reference. The photoluminescence (PL) spectra

were measured by a Perkin-Elmer LS55 fluorescence spectrophotometer with excitation wavelength of 320 nm at room temperature. Time-resolved transient PL decay curves were obtained using a FLS920 fluorescence lifetime spectrophotometer (Edinburgh Instruments, U.K.) with 320 nm excitation wavelength and 610 nm emission wavelength. Electron spin-resonance spectroscopy (ESR) was collected on a magnettech MS-5000 instrument. The zeta potentials of as-obtained samples were measured using a zeta potential analyzer (Malvern, Zetasizer Nano, ZS90) at 293K. The photodegrading intermediate products in reaction solution were detected by Agilent 1100 series high-performance liquid chromatography/mass spectrometry detector (HPLC/MSD Trap/VL). The total organic carbon (TOC) tests were carried out on a Sievers InnovOx Laboratory TOC Analyzer. The ICP (inductively coupled plasma) test was carried on ICP-6000 instrument.

S1.4 Photocatalytic degradation of TC, ENR and MO

20 mg of various samples were dispersed into 20 mL of TC (20 ppm), ENR (20 ppm) or MO (20 ppm; pH=1) aqueous solution [79]. Before light irradiation, the mixed solution was placed in the dark for a period of time to make the adsorption-desorption equilibrium between TC, ENR and MO molecules and catalyst. Under continuously stirring, the solution was irradiated with a 300 W xenon lamp with a light intensity of 100 mW/cm^2 (with a 420 nm filter). for photocatalytic reaction. The vertical distance between the mixture and the lamp is about 12 cm. At a given time, about 2 mL suspension was removed and collected by centrifugation to completely remove the catalyst powder. The supernatant was determined at the maximum absorption

wavelength of 357 nm for TC, 322 nm for ENR and 506 nm for MO.

79. H, D.L.; C, Y.F.; S, Y.; Zhong, L.; Huang, H. Synthesis of ternary g-C_3N_4/Ag/-FeOOH photocatalyst: an integrated heterogeneous fenton-like system for effectively degradation of azo dye methyl orange under visible light, Appl. Surf. Sci. **2017**, 425, 862-872.

S1.5 Zeta-potential measurement

Typically, each sample powder (5 mg) was dispersed in 100 mL of water by ultrasonication for 30 min. The zeta potential of each sample was measured for three times by testing the suspension with the initial pH value. In order to obtain the isoelectric point, the pH of the suspension was then adjusted to several values between 1 and 11 by adding 0.25 M HCl or NaOH solution using an autotitrator (Malvern, MPT-2). Three zeta potential readings were taken at every pH value, and the average zeta potential at a certain pH value was plotted against the pH value. The pH value where the zeta potential was zero was taken as the IEP.

S1.6 Active species trapping experiment and ESR measurements

For degradation of TC: Triethanolamine (TEOA), isopropanol (IPA) and 4-hydroxy-TEMPO were used respectively as hole (h^+) scavenger, hydroxyl radical (·OH) scavenger and superoxide radical ($\cdot O_2^-$) scavenger to investigate the active species in the photodegradation process of TC. In general, 20 mg of 10% Ag/PT with various scavenger was dispersed in 20 mL of 40 ppm TC aqueous solution, and the following process was the same as the degradation process.

The ESR measurements with 5,5-dimethyl-1-pyrroline N-oxide (DMPO) and TEMPO as the spin trap were adopted to distinguish intermediate species especially such as superoxide anion radicals ($\cdot O_2^-$), hydroxyl free radicals (·OH) and holes (h^+)

generated during the reaction. Electron spin-resonance spectroscopy (ESR) for $\cdot O_2^-$, $\cdot OH$ and h^+ was determined in dark and under visible light irradiation (methanol solution volume: 1.0 mL; sample: 3 mg; DMPO/TEMPO: 0.2 mL (1 M)).

S1.7 Electrochemical measurements

The electrochemical characterizations were conducted with a CHI760E electrochemical workstation (Shanghai ChenHua Instrument Co., Ltd., China) with a conventional three-electrode configuration in a quartz cell. For photoelectrochemical measurement, an Hg/Hg_2Cl_2 electrode and Pt foil were used as the reference electrode and counter electrode, respectively. A 0.5 M Na_2SO_4 aqueous solution was used as electrolyte. A 300 W Xe lamp was employed to provide light source. Typically, the working electrodes were prepared as follows: 20 mg of various catalyst was dispersed into 3 mL of ethanol with sonication for 20 min. Then, 0.5 mL of the above solution was uniformly dropped onto a 1×3 cm^2 FTO glass substrate (an active area of ca. 2 cm^2). Finally, the as-prepared electrodes were dried at 70 ^oC for 4 h to obtain the working electrodes. The electrochemical impedance spectroscopy (EIS) data was collected in 0.1 M KCl solution containing 5 mM $Fe(CN)_6^{3-/4-}$ with a frequency range from 0.01 Hz to 10 kHz at 0.1 V. And the equivalent circuit was obtained using Zview software.

The Mott-Schottky test was carried out with a standard three-electrode system using the prepared glass electrode as the working electrode, a Pt wire as a counter electrode, Hg/Hg_2Cl_2 electrode as a reference electrode, respectively. A 0.5 M Na_2SO_4 solution was applied as the electrolyte. Mott-Schottky plots were measured at a fixed frequency of 1000 Hz and an amplitude of 50 mV without light illumination.

S1.8 TRPL spectrum analysis

The time-resolved fluorescence decay (TRPL) spectrum was used to further confirm the photoelectron and hole transfer behavior. The two exponential decay model is used to fit the corresponding time-resolved spectrum, the formulas are as follows [Eqs.(S1) and (S2)]:

$$R(t) = \sum_{i=1,2} A_i e^{-t/t_i} \tag{S1}$$

$R(t)$ is the normalised emission intensity; t is the time after the pulsed laser excitation; t_i are the respective decay lifetimes; A_i are the corresponding weight factors.

$$\langle \tau \rangle = \frac{\sum_{i=1,2} A_i \tau_i^2}{\sum_{i=1,2} A_i \tau_i} \tag{S2}$$

2. Figures

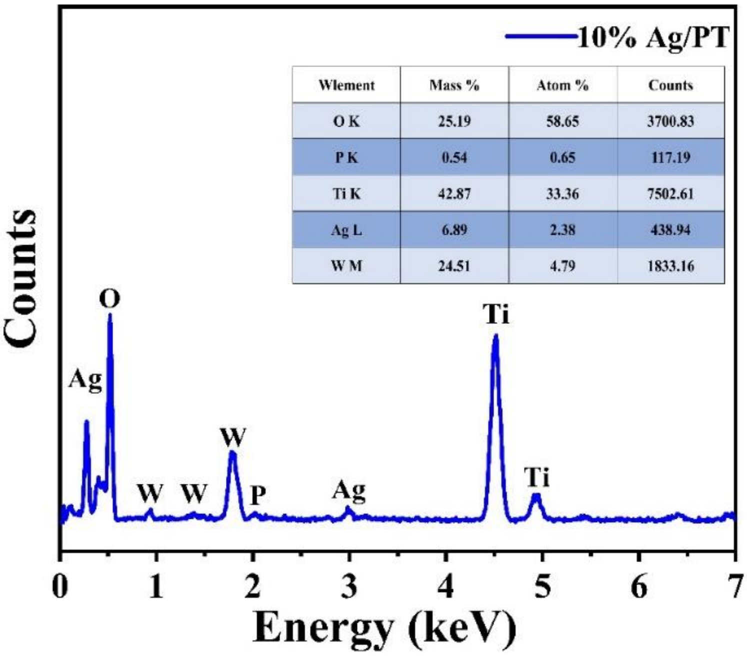

Figure S1. EDX data of 10% Ag/PT sample.

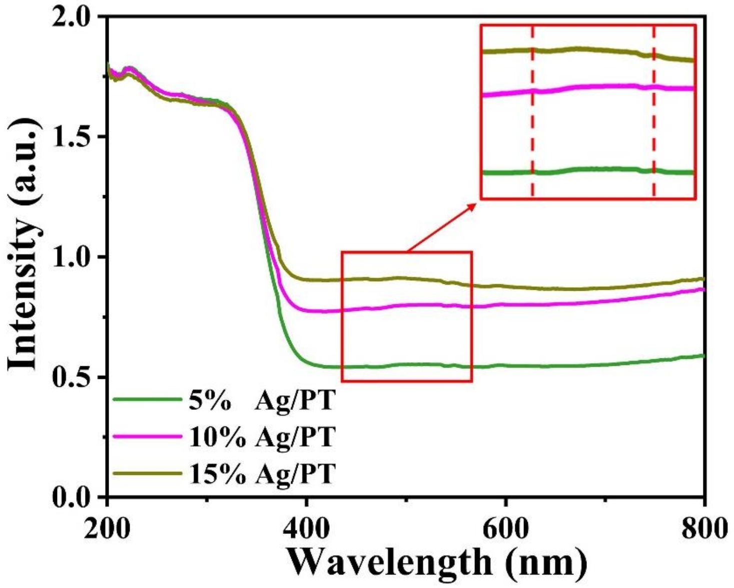

Figure S2. UV-Vis absorption spectra of 5%, 10% and 15% Ag/PT samples.

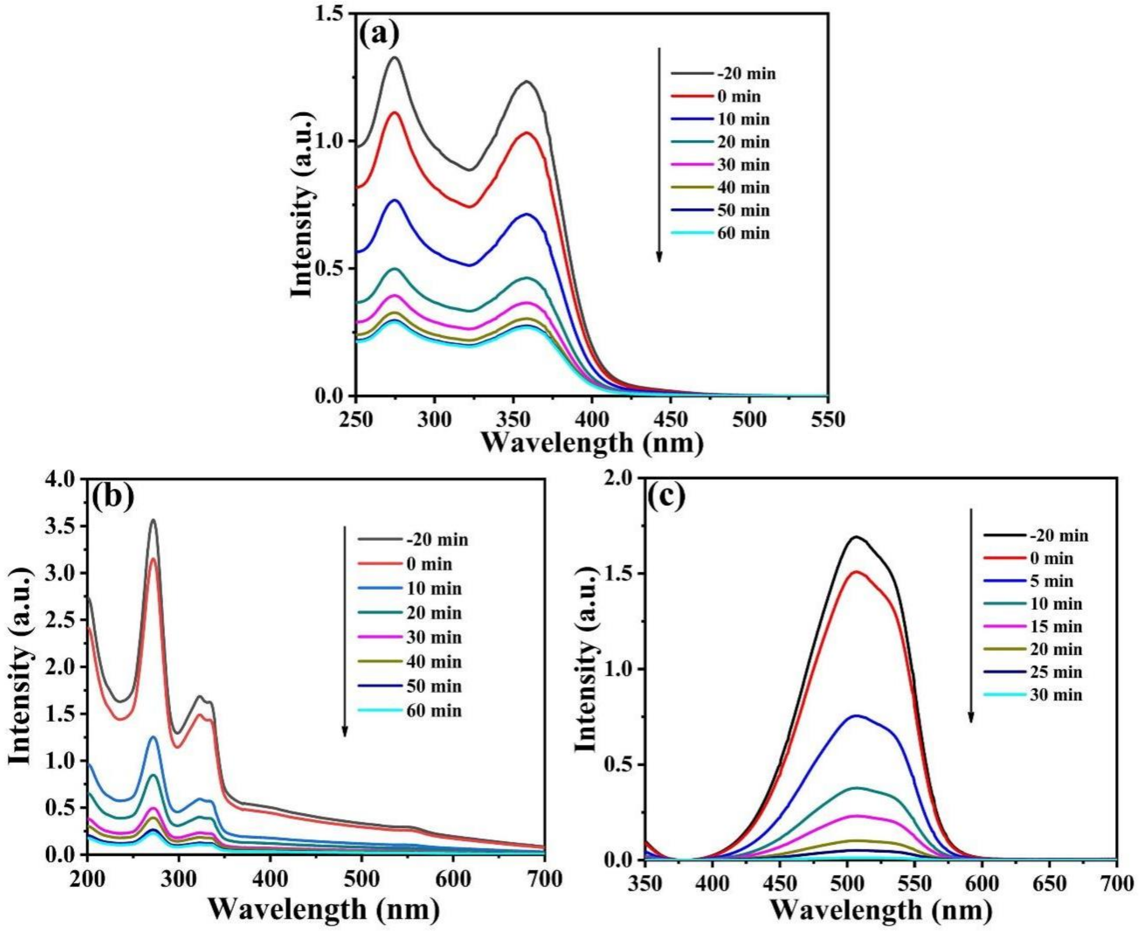

Figure S3. The profiles of photocatalytic degradation of TC (a), ENR (b) and MO (c) by 10% Ag/PT under visible-light irradiation ($\lambda > 420$ nm).

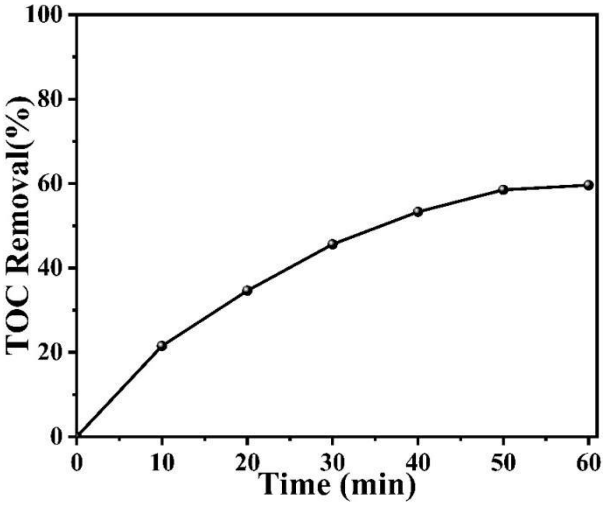

Figure S4. The TOC removal (%) for TC degradation by 10% Ag/PT sample.

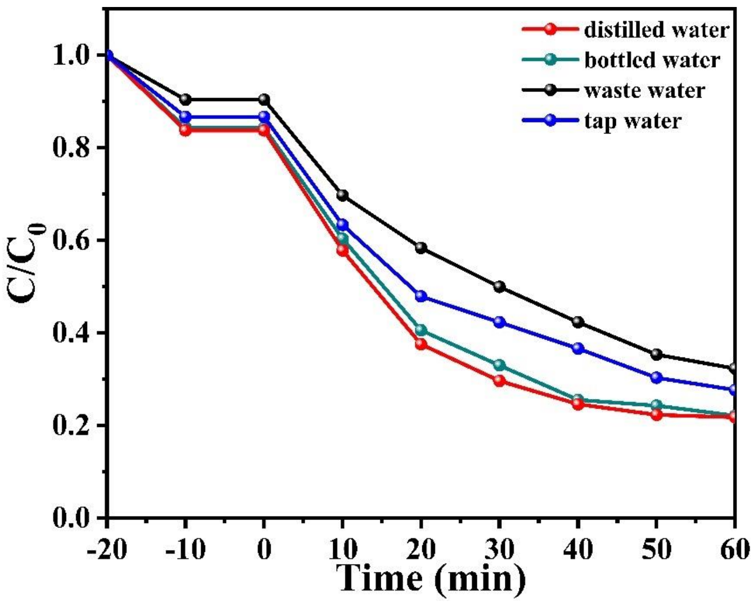

Figure S5. Photodegradation of TC with 10% Ag/PT in diverse water quality (catalyst amount: 20 mg; TC: 20 mL of 20 ppm).

Influence of water quality. Figure S5 reveals the effect of deionized water, bottled water, waste water and laboratory tap water on the TC degradation rate. The order of catalytic activity was as follows: deionized water (78.19%) > bottled water (77.86%) > tap water (72.32%) > waste water (67.67%). The inhibition of catalytic activity in waste water and tap water may be caused by competing substances in actual water, such as inorganic ions and dissolved organic matter [80].

80. Liang, J.X.; Hou, Y.P.; Zhu, H.X.; Xiong, J.H.; Huang, W.Y.; Yu, Z.B.; Wang, S.F. Levofloxacin degradation performance and mechanism in the novel electro-Fenton system constructed with vanadium oxide electrodes under neutral pH, *Chem. Eng. J.* **2021**, 433, 133574.

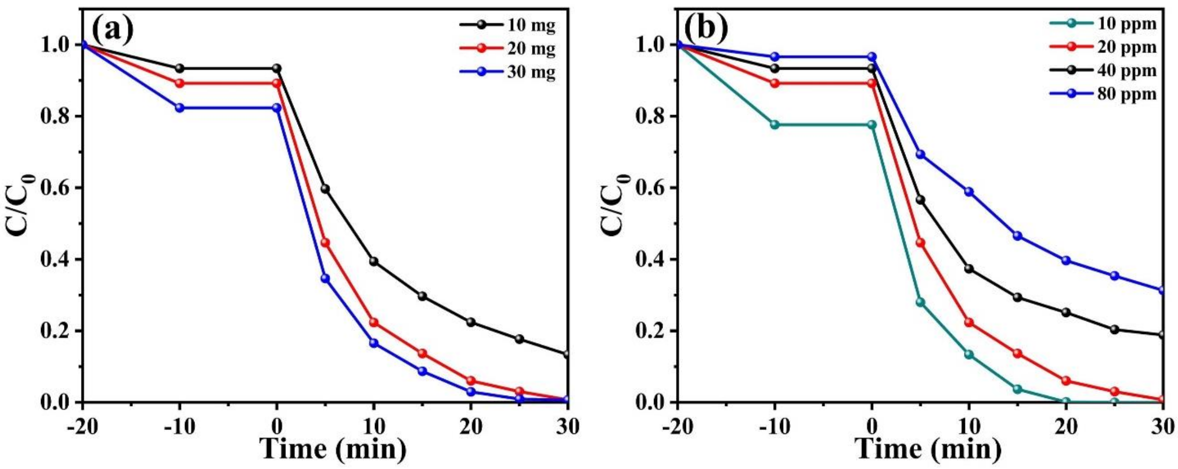

Figure S6. Degradation of MO with 10% Ag/PT with various conditions: (a) Diverse catalyst amount (MO: 20 mL of 20 ppm; pH = 1) and (b) Different concentration of MO (MO: 20 mL; pH = 1; catalyst amount: 20 mg).

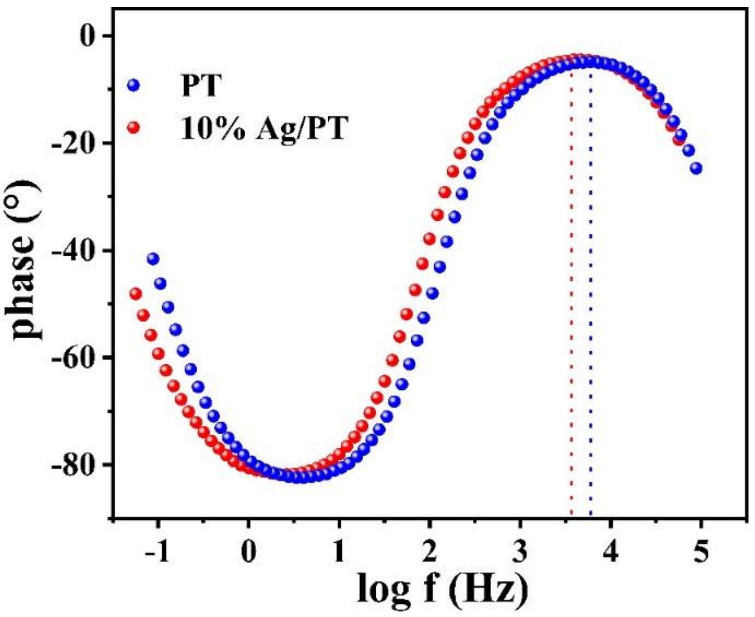

Figure S7. The Bode plots of PT and 10% Ag/PT composite.

The electron-hole separation situation of different samples was also compared via the Bode plots (Fig. S4). The electron lifetime (τ_r) can be calculated from the following equation:

$$\tau_r = \frac{1}{2\pi f_{peak}} \quad \text{(S3)}$$

where f_{peak} is the frequency that is located at the mid-frequency (1-100 Hz) range. Therefore, the lifetime of the photogenerated electrons is in reverse related to f_{peak}. Fig.S4 presents lower peak frequency for PT and 10% Ag/PT composite, indicating the high lifetime of photogenerated electrons for 10% Ag/PT in comparison to PT. The result was in great accord with the results of photocurrent responses.

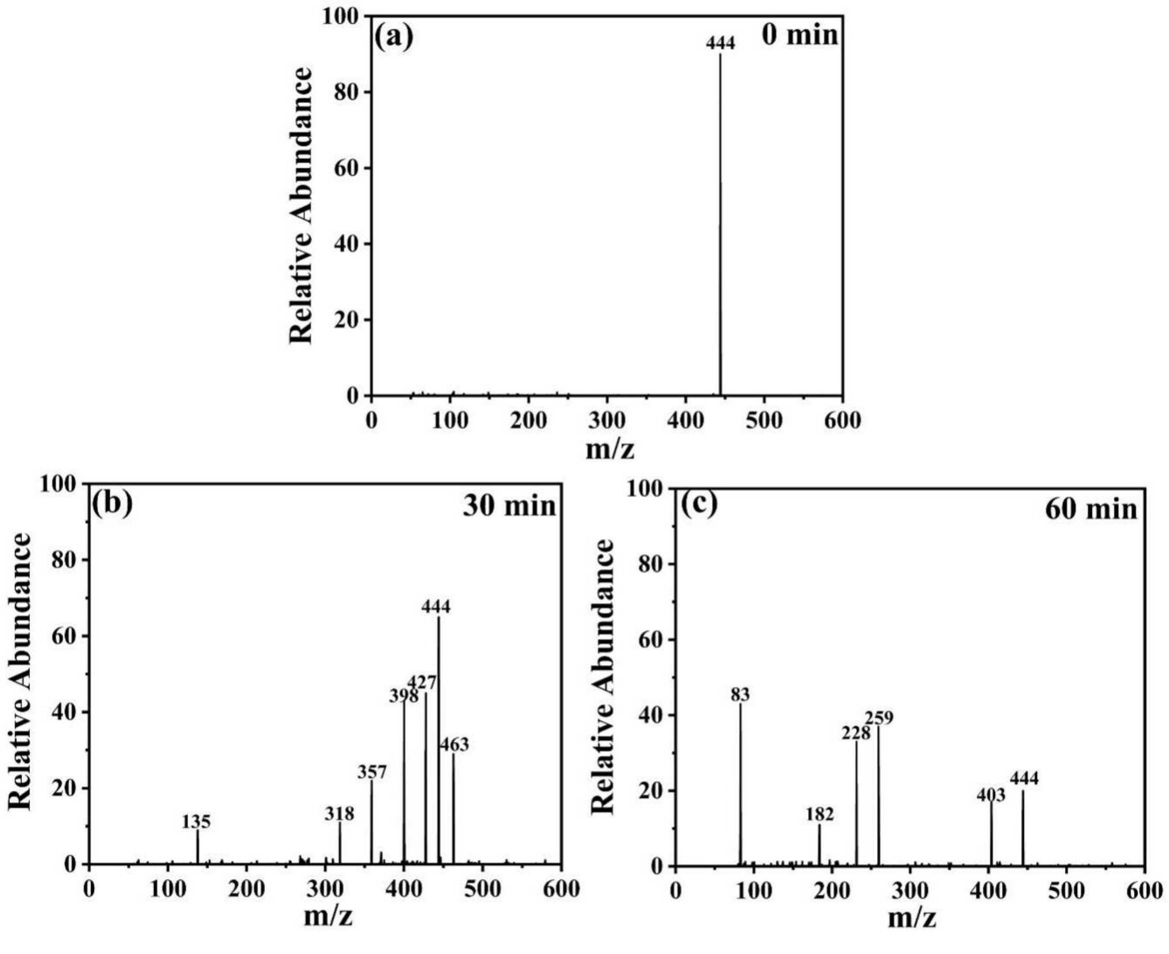

Figure S8. The main intermediate products generated during the photocatalytic TC degradation process: (a) 0 min; (b) 30 min; (c) 60 min with 10% Ag/PT as catalyst.

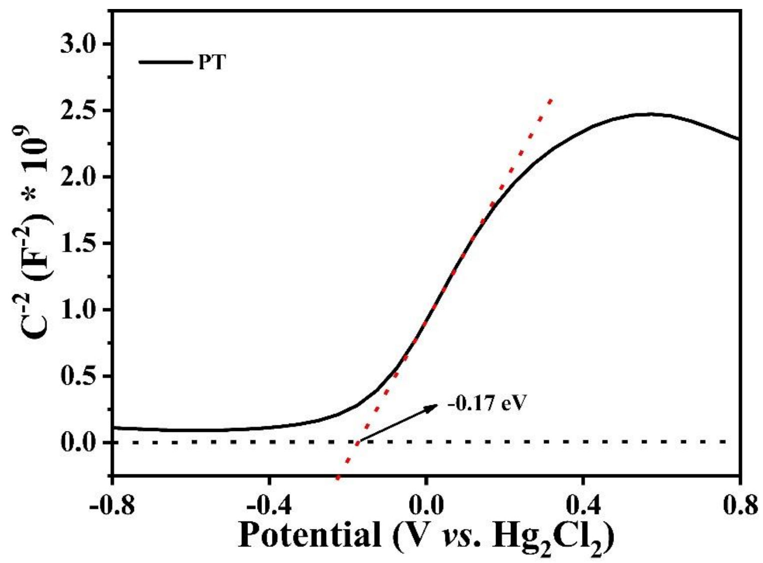

Figure S9. The E_{fb} of PT (V *vs*. Hg/Hg_2Cl_2).

3. Tables

Table S1. The comparison of TC degradation activity of 10% Ag/PT with previous literatures.

Photocatalyst	Concentration (mg L^{-1})	Dosage (g L^{-1})	Time (min)	Removal (%)	Light source	Reference
TiO_2	20	1	30	85.0	500 W Xe lamp; λ > 420 nm	81
Mn_2O_3/Bi_2O_3	45	0.2	100	73.34	300 W Xe lamp; λ > 357 nm	82
ZnO@ZIF-8	20	0.5	90	91.2	300 W Xe lamp; λ > 420 nm	83
1.0 wt% MoS_2/BiOBr	20	0.5	120	68	300 W Xe lamp; λ > 400 nm	84
B-TiO_2	20	0.2	270	86.40	1000 W Xe lamp; λ > 400 nm	85
NiO/g-C_3N_4	20	0.1	60	90.1	300 W Xe lamp; λ ≥ 420 nm	86
g-C_3N_4/CdS	20	0.2	60	81	300 W Xe lamp; λ ≥ 400 nm	87
Fe/Sn-TiO_2	10	0.3	60	92	300 W Xe	88

					lamp; $\lambda > 420$ nm	
BiOBr/rGO	10	0.125	140	100.0	300 W Xe lamp; λ < 400 nm	89
CuO/Fe_3O_4/GO	30	0.2	150	97.3	300 W Xe lamp; $\lambda > 420$ nm	90
B-TiO_2/$BiVO_4$	20	0.5	120	89.3	300 W Xe lamp; $\lambda > 400$ nm	91
Ag_2CO_3/$Bi_4O_5I_2$/ g-C_3N_4	20	0.6	30	82.2	300 W Xe lamp; $\lambda > 420$ nm	92
Mn_yO_x	50	0.4	150	96.0	24.8W LED lamp; 450-460 nm	93
hierarchical	100	0.5	30	81.7	300 W Xe lamp; $\lambda > 420$ nm	94
$K_5CoW_{12}O_{40}$/TiO_2	50	1	120	97.0	300 W Xe lamp; $\lambda > 420$ nm	95
**Ag/PT**	**20**	**1**	**60**	**78.16**	**300 W Xe lamp; $\lambda > 420$ nm**	**This work**

References

81. Gong, Y.N.; Wang, Y.; Tang, M.M.; Zhang, H.; Wu, P.; Liu, C.J.; He, J.; Jiang, W. A two-

step process coupling photocatalysis with adsorption to treat tetracycline-Copper(II) hybrid wastewaters, degradation mechanism, pathways and biotoxicity evaluation. *J. Water. Process. Eng.* **2022**, 47, 102710.

82. Chen, L.J.; Li, Y.H.; Zhang, J.W.; Li, M.X.; Yin, W.Y.; Chen, X. Oxidative degradation of tetracycline hydrochloride by Mn_2O_3/Bi_2O_3 photocatalysis activated peroxymonosulfate. *Inorg. Chem. Commun.* **2022**, 140, 109414.

83. Zhang, X.M.; Wang, H.; Gao, M.M.; Zhao, P.F.; Xia, W.L.; Yang, R.L.; Huang, Y.C.; Wang, L.; Liu, M.X.; Wei, T.; Wang, L.; Yao, R.X.; Li, X.; Fan, Z.J. Template-directed synthesis of pomegranate-shaped zinc oxide@zeolitic imidazolate framework for visible light photocatalytic degradation of tetracycline. *Chemosphere* **2022**, 294, 133782.

84. Yin, W.Q.; Cao, X.J.; Wang, B.; Jiang, Q.; Chen, Z.G.; Xia, J.X. In-situ synthesis of MoS_2/BiOBr material via mechanical ball milling for boosted photocatalytic degradation pollutants performance. *ChemistrySelect* **2021**, 6, 928-936.

85. Wu, S.Q.; Li, X.Y.; Tian, Y.Q.; Lin, Y.; Hu, Y.H. Excellent photocatalytic degradation of tetracycline over black anatase-TiO_2 under visible light. *Chem. Eng. J.* **2021**, 406, 126747.

86. Li, S.Y.; Tang, Y.W.; Wang, M.; Kang, J.; Jin, C.Y.; Liu, J.Y. Li, Z.L.; Zhu, J.W. NiO/g-C_3N_4 2D/2D heterojunction catalyst as efficient peroxymonosulfate activators toward tetracycline degradation: Characterization, performance and mechanism. *J. Alloys Compd.* **2021**, 880, 160547.

87. Shen, X.F.; Zhang, Y.; Shi, Z.; Shan, S.D.; Liu, J.S.; Zhang, L.S. Construction of C_3N_4/CdS nanojunctions on carbon fiber cloth as a filter-membrane-shaped photocatalyst for degrading flowing wastewater. *J. Alloys Compd.* **2021**, 851, 156743.

88. Ghoreishian, S.M.; Ranjith, K.S.; Lee, H.; Park, B.; Norouzi, M.; Nikoo, S.Z.; Kim, W.S.; Han, Y.K.; Huh, Y.S. Tuning the phase composition of 1D TiO_2 by Fe/Sn co-doping strategy for enhanced visible-light-driven photocatalytic and photoelectrochemical performances. *J. Alloys Compd.* **2021**, 851, 156826.

89. Jiang, H.; Wang, Q.; Chen, P.; Zheng, H.; Shi, J.; Shu, H.; Liu, Y. Photocatalytic degradation of tetracycline by using a regenerable (Bi)BiOBr/rGO composite. *J. Clean. Prod.* **2022**, 339, 130771.

90. Zhu, L.; Zhou, Y.; Fei, L.; Cheng, X.; Zhu, X.; Deng, L.; Ma, X. Z-scheme CuO/Fe_3O_4/GO

heterojunction photocatalyst: Enhanced photocatalytic performance for elimination of tetracycline. *Chemosphere* **2022**, 309, 136721.

91. Wu, C.; Dai, J.; Ma, J.; Zhang, T.; Qiang, L.; Xue, J. Mechanistic study of B-TiO_2/$BiVO_4$ S-scheme heterojunction photocatalyst for tetracycline hydrochloride removal and H_2 production. *Sep. Purif. Technol.* **2023**, 312, 123398.

92. Chen, Z.J.; Guo, H.; Liu, H.Y.; Niu, C.G.; Huang, D.W.; Yang, Y.Y.; Liang, C.; Li, L.; Li, J.C. Construction of dual S-scheme Ag_2CO_3/$Bi_4O_5I_2$/g-C_3N_4 heterostructure photocatalyst with enhanced visible-light photocatalytic degradation for tetracycline. *Chem. Eng. J.* **2022,** 438, 135471.

93. Cestaro, R.; Philippe, L.; Serrà, A.; Gómez, E.; Schmutz, P. Electrodeposited manganese oxides as efficient photocatalyst for the degradation of tetracycline antibiotics pollutant. *Chem. Eng. J.* **2023**, 462, 142202.

94. Wu, Z.; Wang, M.; Bai, Y.; Song, H.; Lv, J.; Mo, X.; Li, X.; Lin, Z. Upcycling of nickel iron slags to hierarchical self-assembled flower-like photocatalysts for highly efficient degradation of high-concentration tetracycline. *Chem. Eng. J.* **2023**, 464, 142532.

95. Mahmoodi, M.; Rafiee, E.; Eavani, S. Photocatalytic removal of toxic dyes, liquorice and tetracycline wastewaters by a mesoporous photocatalyst under irradiation of different lamps and sunlight. *J. Environ. Manage.* **2022**, 313, 115023.

Table S2. The comparison of ENR degradation activity of 10% Ag/PT with previous literatures.

Photocatalyst	Concentration (mg L^{-1})	Dosage (g L^{-1})	Time (min)	Removal (%)	Light source	Reference
BN/$BiPO_4$	10	0.33	120	91.5	250 W Hg lamp ; λ < 400 nm	96

NIR GC	50	1	30	99.5	500 W Xe lamp; λ > 420 nm	97
$g\text{-}C_3N_4$/ TiO_2	10	1	60	98.5	350 W Xe lamp; λ > 420 nm	98
Ag_2O/CeO_2	10	1	120	87.1	300 W Xe lamp; λ > 420 nm	99
$Cd_{0.5}Zn_{0.5}S/Bi_2MoO_6$	10	0.16	40	76.4	300W Hg lamp; λ > 420 nm	100
Cs_xWO_3/BiOI	20	0.5	60	96.0	300 W Xe lamp; λ > 420 nm	101
BC/CN	10	1	480	90.0	500 W Xe lamp; λ > 420 nm	102
$GaOOH/ZnBiTaO_5$	10	1	60	58.3	500 W mercury lamp; λ < 400 nm	103
Magnox/UVC	25	1	120	99.5	300 W Xe lamp; λ ≥ 400 nm	104
$TiO_2/g\text{-}C_3N_4$	4	1	60	91.66	350 W Xe lamp; λ > 420 nm	105
$GaOOH/ZnBiNbO_5$	10	1	60	65.31	500 W	106

					mercury lamp; λ < 400 nm	
Fe_3O_4@TiO_2-GO	2	0.5	240	96.0	500 W Xe lamp; λ>420 nm	107
CdS/CuAg	20	0.02	45	100.0	400 W Hg lamp; λ >420 nm	108
Er_2FeSbO_7/$BiTiSbO_6$	10	1	120	91.5	300 W Xe lamp; 420 nm>λ	109
Pt-$Bi_4V_2O_{11}$	40	1	150	98.0	400 W Xe lamp; λ ≥ 420 nm	110
**Ag/PT**	**20**	**1**	**60**	**93.65**	**300 W Xe lamp; λ > 420 nm**	**This work**

References

96. Chen, Z.G.; Chen, X.L.; Di, J.; Liu, Y.L.; Yin, S.; Xia, J.X.; Li, H.M. Graphene-like boron nitride modified bismuth phosphate materials for boosting photocatalytic degradation of enrofloxacin. *J. Colloid. Interf. Sci.* **2017**, 492, 51–60.

97. Liu, Y.C.; Li, G.B.; Wang, D.; Zhong, Z.C.; Hu, K.B.; Zhang, C.Q.; Hu, G.P.; Li, X.W.; Wan Y.H. Lanthanide-doped upconversion glass-ceramic photocatalyst fabricated from fluorine-containing waste for the degradation of organic pollutants. *J. Colloid. Interf. Sci.* **2023**, 638, 461-473.

98. Huang, J.X.; Li, D.G.; Li, R.B.; Chen, P.; Zhang, Q.X.; Liu, H.J.; Lv, W.Y.; Liu, G.G.; Feng, Y.P. One-step synthesis of phosphorus/oxygen co-doped g-C_3N_4/anatase TiO_2 Z-scheme

photocatalyst for significantly enhanced visible-light photocatalysis degradation of enrofloxacin. *J. Hazard. Mater.* **2020**, 386, 12.

99. Wen, X.J.; Niu, C.G.; Zhang, L.; Liang, C.; Zeng, G.M. A novel Ag_2O/CeO_2 heterojunction photocatalysts for photocatalytic degradation of enrofloxacin: possible degradation pathways; mineralization activity and an in depth mechanism insight. *Appl. Catal. B-Environ.* **2018**, 221, 701-714.

100. Cai, M.J.; Liu, Y.P.; Wang, C.C.; Lin, W.; Li, S.J. Novel $Cd_{0.5}Zn_{0.5}S/Bi_2MoO_6$ S-scheme heterojunction for boosting the photodegradation of antibiotic enrofloxacin: Degradation pathway; mechanism and toxicity assessment. *Sep. Purif. Technol.* **2023**, 304, 11.

101. Li, T.C.; Liu, J.X.; Shi, F.; Zhang, H.Y.; Zhang, H.J.; Ma, C.C.; Wasim, M. A novel S-type $CsxWO_3$/BiOI heterojunction photocatalyst constructed in graphene aerogel with high degradation efficiency for enrofloxacin: Degradation mechanism and DFT calculation. *J. Environ. Chem. Eng.* **2023**, 11, 109301.

102. Xiao, L.Q.; Zhang, S.Y.; Chen, B.Q.; Wu, P.P.; Feng, N.D.; Deng, F.; Wang, Z. Visible-light photocatalysis degradation of enrofloxacin by crawfish shell biochar combined with $g-C_3N_4$: Effects and mechanisms. *J. Environ. Chem. Eng.* **2023**, 11, 109693.

103. Huang, P.Q.; Luan, J.F. Synthesis of a $GaOOH/ZnBiTaO_5$ heterojunction photocatalyst with enhanced photocatalytic performance toward enrofloxacin. *RSC Adv.* **2020**, 10, 4286-4292.

104. Sciscenko, I.; Mestre, S.; Climent, J.; Valero, F.; Escudero-Onate, C.; Oller I.; Arques, A. Magnetic Photocatalyst for Wastewater Tertiary Treatment at Pilot Plant Scale: Disinfection and Enrofloxacin Abatement. *Water* **2021**, 13, 12.

105. Su, Y.H.; Chen, P.; Wang, F.L.; Zhang, Q.X.; Chen, T.S.; Wang, Y.F.; Yao, K.; Lv, W.Y.; Liu, G.G. Decoration of $TiO_2/g-C_3N_4$ Z-scheme by carbon dots as a novel photocatalyst with improved visible-light photocatalytic performance for the degradation of enrofloxacin. *RSC Adv.* **2017**, 7, 34096-34103.

106. Huang, P.Q.; Luan, J.F. Dispersed GaOOH rods loaded on the surface of $ZnBiNbO_5$ particles with enhanced photocatalytic activity toward enrofloxacin. *RSC Adv.* **2019**, 9, 32027-32033.

107. Yu, Y.Q.; Yan, L.; Cheng, J.M.; Jing, C.Y. Mechanistic insights into TiO_2 thickness in

Fe_3O_4@TiO_2-GO composites for enrofloxacin photodegradation. *Chem. Eng. J.* **2017**, 325, 647-654.

108. Mahjoub, A.R.; Rahmani, H.; Khazaee, Z. Bimetallic CuAg alloyed nanoparticles anchored on CdS nanorods for the photocatalytic degradation of enrofloxacin. *ACS Appl. Nano. Mater.* **2023**, 6, 4554-4566.

109. Luan, J.F.; Liu, W.L.; Yao, Y.; Ma, B.B.; Niu, B.W.; Yang, G.M.; Wei, Z.J. Synthesis and Property Examination of Er_2FeSbO_7/$BiTiSbO_6$ Heterojunction Composite Catalyst and Light-Catalyzed Retrogradation of Enrofloxacin in Pharmaceutical Waste Water under Visible Light Irradiation. *Materials* **2022**, 15, 26.

110. Zhao, Y.J.; Liu, X.T.; Gu, S.N.; Liu, J.M. Enhanced photocatalytic performance of rhodamine B and enrofloxacin by Pt loaded $Bi_4V_2O_{11}$: boosted separation of charge carriers; additional superoxide radical production; and the photocatalytic mechanism. *RSC Adv.* **2021**, 11, 9746-9755.

Table S3. The comparison of MO degradation activity of 10% Ag/PT with previous literatures.

Photocatalyst	Concentration ($mg L^{-1}$)	Dosage ($g L^{-1}$)	Time (min)	Removal (%)	Light source	Reference
Au/TiO_2	10	1	30	74.2	300 W Xe lamp; $\lambda > 420$ nm	111
AC-Bi_5O_7I	10	0.5	240	96.8	150 W Xe lamp; $\lambda > 420$ nm	112
$CoNiO_2$/$BFeO_3$/NiS	25	1	160	99.9	300 W Xe lamp; $\lambda > 400$	113

					nm	
$Bi_2WO_6/ZnIn_2S$	10	1	60	97.5	300 W Xe lamp; λ > 420 nm	114
ms-$BiVO_4$	20	1	120	89.5	450 W Xe lamp; λ > 420 nm	115
$Ti_3C_2/eTiO_2$	30	0.58	40	99.0	320 W Xe lamp; λ > 420 nm	116
TiO_2/Fe_2O_3/CNTs	30	0.67	240	80.0	300 W Xe lamp; λ > 400 nm	117
Eu^{2+}, Dy^{3+}/ZnO	38.4	1	30	99.9	160 W Xe lamp; λ > 200 nm	118
c-Cu_2O	5	0.5	100	92.8	300 W Xe lamp; λ ≥ 420 nm	119
TiO_2–Mo	25	1	120	90.0	150 W Xe lamp; λ > 420 nm	120
[Co(OH-Cor)(PPh_3)]/TiO_2	10	0.5	150	86.5	30 W Xe lamp; λ > 420 nm	121
$CsPbBr_3/Cs_4PbBr_6$	20	1	25	99.9	300 W Xe lamp; λ > 420 nm	122
GA/BiOI	20	0.4	120	93.1	5×55W Xe	123

					lamp; λ > 400 nm	
Pt/CeO_2	20	0.3	200	75.8	500 W Xe lamp; λ > 420 nm	124
Zn-PB/CS	10	0.5	550	94.2	40 W Xe lamp; λ > 400 nm	125
**Ag/PT**	**20**	**1**	**40**	**99.79**	**300 W Xe lamp; λ > 420 nm**	**This work**

References

111. Tian, J.L.; Wu, S.; Liu, S.X.; Zhang, W. Photothermal enhancement of highly efficient photocatalysis with bioinspired thermal radiation balance characteristics. *Appl. Surf. Sci.* **2022**, 592, 153304.

112. Sane, P.K.; Rakte, D.; Tambat, S.; Bhalinge, R.; Sontakke, S.M.; Nemade, P. Enhancing solar photocatalytic activity of Bi_5O_7I photocatalyst with activated carbon heterojunction. *Adv. Powder. Technol.* **2022**, 33, 103357.

113.Harikumar, B.; Okla, M.K.; Alaraidh, I.A.; Mohebaldi, A.; Soufa, W.; Abdel-Maksoud, M.A.; Aufy, M.; Thomas, A.M.; Raju, L.L.; Khan, S. Robust visible light active $CoNiO_2$–$BiFeO_3$–NiS ternary nanocomposite for photo-fenton degradation of rhodamine B and methyl orange: kinetics; degradation pathway and toxicity assessment. *J. Environ. Manage.* **2022**, 317, 115321.

114. Bi, H.F.; Liu, J.S.; Wu, Z.Y.; Zhu, K.J.; Suo, H.; Lv, X.L.; Fu, Y.L.; Jian, R.; Sun, Z.B. Construction of Bi_2WO_6/$ZnIn_2S_4$ with Z-scheme structure for efficient photocatalytic performance. *Chem. Phys. Lett.* **2021**, 769, 138449.

115. Van, N.U.; Thuy, N.P.; Hanh, V.N.; Loan, D.T.; Vuong, D.B.; Thao, T.T. Low-temperature designing of $BiVO_4$ nanocubes with coexposed {010}/{110} facets for solar light

photocatalytic degradation of methyl orange and diazinon. *Inorg. Chem. Commun.* **2022**, 136, 109136.

116. Hieu, V.Q.; Phung, T.K; Nguyen, T.; Khan, A.; Doan, V.D.; Tran, V.A.; Le, V.T. Photocatalytic degradation of methyl orange dye by Ti_3C_2-eTiO_2 heterojunction under solar light. *Chemosphere* **2021**, 276, 130154.

117. Wang, L.; Li, T.; Tao, L.L.; Lei, H.W.; Ma, P.Y.; Liu, J. A novel copper-doped porous carbon nanospheres film prepared by onestep ultrasonic spray pyrolytic of sugar for photocatalytic degradation of methyl orange. *Process. Saf. Environ.* **2022**, 158, 79–86.

118. Menon, S.G.; Bedyala, A.K.; Pathakc, T.; Kumara, V.; Swart, H.C. $Sr_4Al_{14}O_{25}$: Eu^{2+}, Dy^{3+}@ZnO nanocomposites as highly efficient visible light photocatalysts for the degradation of aqueous methyl orange. *J. Alloys Compd.* **2021**, 860, 158370.

119. Li, J.W.; He, M.Z.; Yan, J.K.; Liu, J.H.; Zhang, J.X.; Ma, J.J. Room temperature engineering crystal facet of Cu_2O for photocatalytic degradation of methyl orange. *Nanomaterials* **2022**, 10, 1697.

120. Kanakaraju, D.; Jasni, M.A.A.; Lim, Y.C. A highly photoresponsive and efficient molybdenum-modifiedtitanium dioxide photocatalyst for the degradation of methyl orange. *Int. J. Environ. Sci. Te.* **2022**, 19, 5579–5594.

121. Lu, G.F.; Liu, X.D.; Zhang, P.; Xu, S.T.; Gao, Y.J.; Yu, S.Y. Preparation and Photocatalytic Studies on Nanocomposites of 4-Hydroxylphenyl-Substituted Corrole/TiO_2 towards Methyl Orange Photodegradation. *ChemistrySelect* **2021**, 6, 6841-6846.

122. Kourab, P.; Mukherjee, S.P. $CsPbBr_3$/Cs_4PbBr_6 perovskite@COF nanocomposites for visible-light-driven photocatalytic applications in water. *J. Mater. Chem. A.* **2021**, 9, 6819-6826.

123. Arumugam, M.; Seralathan, K.; Praserthdam, S.; Tahir, M.P. Synthesis of novel graphene aerogel encapsulated bismuth oxyiodide composite towards effective removal of methyl orange azo-dye under visible light. *Chemosphere* **2022**, 303, 135121.

124. Tang, H.D.; Zhang, W.J.; Meng, Y.; Xie, B.; Ni, Z.M.; Xia, S.J. Investigation onto the performance and mechanism of visible light photodegradation of methyl orange catalyzed by M/CeO_2 (M=Pt; Ag; Au). *Mater. Res. Bull.* **2021**, 144, 111497.

125. Aadnan, I.; Zegaoui, O.; Mragui, A.E.; Silva, J.C.G.E. Physicochemical and

photocatalytic properties under visible light of ZnO-Bentonite/Chitosan hybrid-biocompositefor water remediation. *Nanomaterials* **2022**, 12, 4169-007.

Table S4. Fitted parameters of the TRPL decay profiles.

**Sample**	**τ_1 (ns)**	**A_1 (%)**	**τ_2 (ns)**	**A_2 (%)**	**τ_{ave} (ns)**
PT	0.052	79.88	0.272	20.12	0.18
10% Ag/PT	0.014	-9.9	0.056	109.9	0.06
